# Development of Tunable Rose Bengal-Based nanoGUMBOS as Potential Selective Chemotherapeutic Agents

**DOI:** 10.1021/acsomega.5c05765

**Published:** 2025-11-13

**Authors:** William J. A. Russell, Dylan J. Williams, Daylon G. Douglas, Jannet Kocerha, Rocío L. Pérez

**Affiliations:** Center of Advance Materials Science (CAMS), Department of Biochemistry, Chemistry, and Physics, Georgia Southern University, Statesboro, Georgia 30458, United States

## Abstract

As cancer remains one of the greatest threats to the global population, the development of chemotherapeutic agents that are selective and tunable is of critical importance. Herein, a series of Rose Bengal (RB)-based GUMBOS, a ***g*** roup of ***u*** niform ***m*** aterials ***b*** ased on ***o*** rganic ***s*** alts, was synthesized via counterion exchange between [Na]_2_[RB] and three organic cations: tetrabutylphosphonium bromide [TBP]­[Br], tetraphenylphosphonium chloride [TPP]­[Cl], and 1-dodecyl-3-methylimidazolium chloride [C_12_MIm]­[Cl]. The resulting GUMBOS were characterized through FT-IR, ESI-MS, NMR, and UV–vis spectroscopy. Partition coefficient studies indicated that all GUMBOS exhibited hydrophobic physicochemical characteristics. These hydrophobic RB-based GUMBOS were formed into nanoparticles via the reprecipitation method and exhibited average diameter sizes below 85 nm with moderately negative ζ-potentials all below −30 mV. Permeability through biological membranes was assessed by an *in vitro* PAMPA, revealing enhanced passive diffusion at 10 μM solutions. The cytotoxic activity of the nanoparticles was assessed in A549 lung carcinoma cells as well as noncancerous HEK239 human embryonic kidney cells. The A549 cell line exhibited mainly apoptotic characteristics, with a lower average cell viability of 22.6% from treatment with [C_12_MIm]_2_[RB] nanoGUMBOS. Moreover, the HEK239 cells exhibited a higher viability of 47.1% with the same treatment. The IC50 of the [C_12_MIm]_2_[RB] nanoGUMBOS treatment on cancerous cells was 2.16 μM, with a selectivity index of 2.1. These findings demonstrate the novelty of RB-based nanoGUMBOS as tunable and selective chemotherapeutic agents.

## Graphical abstract

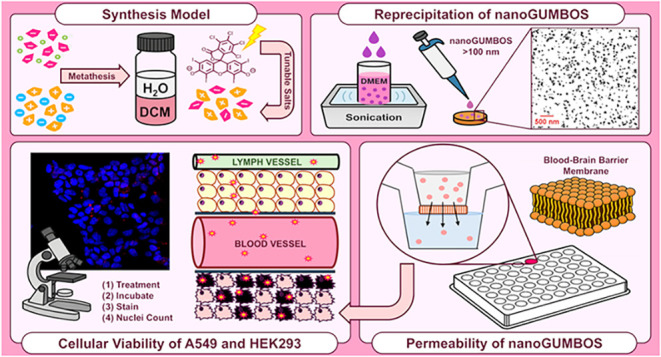

## 1 Introduction

Cancer is characterized by uncontrolled cell proliferation due to dysfunctional biological processes.^1^ As the second leading cause of death worldwide, it significantly impacts human health.^2^ Current chemotherapies often fall short in effectively treating cancer and can disrupt homeostatic functions in noncancerous cells.^3^ The adaptation within such cancers to resist drug inhibition, such as enhanced DNA repair and reduced drug uptake, further complicate treatment due to various mechanisms, including overexpression of efflux pumps and altered cellular metabolism.^4,5^ Systemic toxicity often is not selective enough to effectively improve the quality of life of patients, increasing recovery periods and limiting the prognosis for many patients. This trend toward treatment resistance necessitates a deep understanding of cellular mechanisms and the development of novel drugs that target these pathways in cancerous cells.^6^ Given the metabolic nature of these cellular functions, it is crucial to design drugs that can target energy production, protein synthesis, membrane elasticity, and other vital functions to improve patient care and extend wellness.^2,3,6^

Current cancer studies focus on the complex synthesis of organic compounds that may have the potential to create reactive oxygen species (ROS) or are able to target some functions that tumor progression relies on. However, these types of approaches to potential chemotherapy research face challenges due to the expenses associated with the creation and sustainability of specific synthetic avenues. Thus, it has become increasingly difficult for patients to afford care, especially since chemotherapies require more thorough syntheses and expensive materials. Understanding potential cellular pathways of interest as drug targets is essential in optimizing the design of these potential chemotherapies. The synthesis of novel chemotherapeutic agents must be coupled with a comprehensive mechanistic understanding to improve therapeutic efficacy. Mitochondria play a critical role in cellular metabolism and controlling mechanistic processes that ensure cellular viability.^7^ Cancer cells often manipulate this organelle’s relationship with cellular function to improve its proliferation, often resulting in a hyperpolarization of the mitochondrial membranes. Thus, selective chemotherapy has been proposed to target such susceptible polarization, in addition to the extensive range of chemical groups that could potentially target these differentiations within cancerous cells.^8^ Despite this, many introduced drugs are not biocompatible or have problems selectively permeating through cancerous differentiated membranes.

Nanotechnology is emerging as a promising approach for targeting these cellular pathways with novel chemotherapies.^9–11^ Traditional treatments, such as artificially created nucleic acids, hormonal optimization, and cytotoxic radiation therapies, often fail to selectively inhibit tumor progression.^9,12,13^ One promising application of nanomedicine is to induce the production of ROS, which can trigger cellular autophagy leading to apoptosis.^14,15^ The mitochondrion, which is responsible for producing adenosine triphosphate (ATP) through cellular respiration, is crucial in this process. ROS can disrupt mitochondrial function and cause DNA damage, increasing cancer cell apoptosis through various oxidative stress mechanisms.^7,8^ This process is closely linked to cellular respiration, which requires the use of oxygen as the terminal electron acceptor.^5,7^ ROS will inhibit the functionality of this systemic process, causing DNA damage and increasing rates of cancer cell apoptosis.^8,16^ Additionally, other metabolic pathways such as the Quinone Methide antioxidant pathway, which mitigates oxidative stress, which can be targeted through acetal-based apoptosis.^17,18^ Acetal- and glutathione-mediated inhibition changes the cellular responses in cancer cells, causing the depletion of concentrated glutathione, a chemical that cancer cells adapt to increase when counteracting ROS-induced oxidative stress.^17^ Thus, drugs that are tuned to be selective can induce ROS formation while targeting antioxidant pathways in only the targeted tumor cells.

Xanthene derivatives have been heavily studied as a group of chemicals and have many applications in materials science and medicinal purposes.^19,20^ The dye Rose Bengal is an anionic xanthene dye that has been previously used as a corneal dye and as an antimicrobial agent.^21–23^ RB photodynamic therapy applications are as follows. It has been employed as a photodynamic therapeutic agent for cancer treatment under laser irradiation. Xu et al. employed RB for the synthesis of catalase-RB nanoparticles that showed an increased mitochondrial selectivity and an increased formation of ROS under laser irradiation, suggesting a strong potential of these nanoparticles as a photodynamic therapeutic agent.^24^ Dhillon et al. synthesized an RB-amphiphilic peptide conjugate that was employed as photodynamic therapy for *in vitro* and *in vivo* experiments, demonstrating a higher cytotoxicity under laser irradiation.^25^

***G*** roup ***u*** niform ***m*** aterials ***b*** ased on ***o*** rganic ***s*** alts (GUMBOS) are a class of solid organic salts with similar characteristics to ionic liquids, but with a melting point range between 25 and 250 °C.^26–34^ These compounds have diverse applications ranging from environmental conservation to sensors for protein detection and specialized chemical affinity.^10,26,28,32,33,35,36^ The variability in counterions allows GUMBOS to tune physical or chemical properties for specific needs, applications, and targeting effects against cancer cells and other pathogens.^7,31,37^ Furthermore, these compounds can efficiently form nanoparticles (nanoGUMBOS) through a simple reprecipitation method, facilitating better cellular uptake and allowing for less aggregation *in vivo* due to enhanced permeation and retention (EPR).^26,38,39^ Photosensitive dyes, in particular, have excellent photosensitive properties that can induce ROS production, offering a novel approach to cancer treatment.^40^

Accordingly, RB was synthesized into three different GUMBOS species through a simple metathesis reaction. Furthermore, nanoGUMBOS were synthesized through a simple reprecipitation method to increase the cellular uptake from cancerous cells.^41–43^ Importantly, all experiments in this study were conducted without light activation, and the observed cytotoxic effects represent a novel nonphotoactivated chemotherapeutic mechanism of nanoGUMBOS, which distinguishes this work from traditional photodynamic therapy approaches. Furthermore, nanoparticle formation of these compounds can be metabolized within cancerous cells or other pathogens and initiate acetal-based apoptosis through intrinsic cellular mechanisms.^44^ This specialization of organic cations complexed with [RB] allows for the increased uptake of the nanoparticles to cancerous cells over the noncancerous counterpart.^43^ While traditional chemotherapeutic agents such as doxorubicin and cisplatin show potent cytotoxic effects, they often lack selectivity and cause significant systemic toxicity. RB-based conjugates reported in the literature typically require photoactivation for optimal activity. In contrast, our nanoGUMBOS demonstrate intrinsic activity without photoactivation and show enhanced selectivity indices compared to the parent RB compound, suggesting distinct therapeutic potential that warrants further investigation. Thus, the importance of the characterization and analysis of these GUMBOS and their interactions can help illuminate the effectiveness of their potential chemotherapeutic action.

## 2 Methods and Materials

### 2.1 Materials

All reagents used in this study were employed as received, without further purification. Dichloromethane (DCM) was sourced from VWR BDH Chemicals (Radnor, PA) and Acros Organics (Fair Lawn, NJ), including extra dry grades stabilized over molecular sieves. Methanol was obtained from Honeywell Burdick & Jackson (Muskegon, MI) for LC–MS grade applications and from Fisher Chemical (Fair Lawn, NJ) as certified ACS grade. Acetonitrile (LC–MS grade) was obtained from Honeywell Burdick & Jackson. Dimethyl sulfoxide (DMSO) was sourced from Fisher Chemical (Fair Lawn, NJ). 1-Octanol was obtained from BeanTown Chemical (Hudson, NH). Sodium chloride was obtained from Fisher Science Education (Nazareth, PA) as well as Flinn Scientific (Batavia, IL). Hydrochloric acid (ACS Plus) was obtained from Winkler Ltd. (Canada). Sodium hydroxide was obtained from Restek (Bellefonte, PA). Calcium chloride dihydrate was obtained from Flinn Scientific. Rose Bengal Disodium Salt ([Na]_2_[RB]), tetrabutylphosphonium bromide ([TBP]­[Br]), tetraphenylphosphonium chloride ([TPP]­[Cl]), and 1-dodecyl-3-methylimidazolium chloride ([C_12_MIm]­[Cl]) were all sourced from Sigma–Aldrich (St. Louis, MO). Silver nitrate (AgNO_3_), sodium monobasic phosphate (NaH_2_PO_4_), potassium dibasic phosphate (KHPO_4_), and potassium chloride (KCl) were similarly obtained from Fisher Scientific. Whatman nylon membrane syringe filters (0.2 μm, 47 mm) were purchased from GE Healthcare Life Sciences (Buckinghamshire, UK; made in the USA). Copper TEM grids (3 mm) were obtained from Ted Pella, Inc. (Redding, CA). Ninety-six-well plates and PAMPA donor/receiver plates were obtained from Fisher Scientific. A549 lung cancer cells and HEK293 human embryonic kidney cells were sourced from ATCC (Manassas, VA). Type III deionized water used in all experiments was obtained via an in-laboratory purification system.

### 2.2 Instrumentation

Ultraviolet–visible (UV–vis) absorption was measured between 560 and 562 nm using a Jasco V-750 spectrophotometer for the characterization of GUMBOS and nanoGUMBOS experiments. Fluorescence excitation and emission spectra were recorded using a Jasco FP-8500 fluorescence spectrophotometer. Fourier transform infrared (FT-IR) spectra were collected using a Thermo Scientific Nicolet iS20 instrument with 256 scans and a resolution of 4 cm^–1^. A JEOL JSM 7600F scanning electron microscope (SEM) was employed for surface imaging and analysis of samples. A Mettler Toledo pH meter was used for measuring the pH of the various solutions. Ultrafast liquid chromatography (UFLC) coupled with a SHIMADZU electrospray ionization-mass spectrometer (ESI-MS) system was used for the characterization of the synthesized GUMBOS. ^1^H and ^13^C NMR spectra were collected using a JEOL ECZL-400S NMR spectrometer (400 MHz, CCRL05) equipped with a 24-position autosample changer and autotune probe. Dynamic light scattering (DLS) and ζ-potential measurements were conducted by using a Malvern Zetasizer Nano-Series system. The blood–brain barrier permeability was assessed by using a Bioassay Systems Parallel Artificial Membrane Permeability Assay-BBB Kit. Confocal fluorescence imaging was performed using a Zeiss confocal laser scanning microscope, LSM-710, equipped with an inverted Axio Observer Z1 platform and Airyscan detection. Triple-deionized water was purified by using a Millipore Milli-Q system.

### 2.3 Synthesis of RB-Based GUMBOS

A simple metathesis reaction system was employed to synthesize RB-based GUMBOS. A counterion exchange occurred between the anionic species [Na]_2_[RB] and each of the cationic species: [TBP]­[Br], [TPP]­[Br], or [C_12_MIm]­[Cl]. This reaction was carried out in a two-phase system consisting of a 75:25 mixture of DCM:H_2_O, using 0.1 g of [Na]_2_[RB] and a stoichiometrically equivalent mass of each cationic species. The reaction mixture was stirred continuously at room temperature, protected from light, for 48 h. After incubation, the DCM layer was washed with fresh water to remove by-products (NaBr or NaCl), which was confirmed by the addition of silver nitrate. Once no precipitate was observed, the water layer was discarded and the DCM layer was removed by rotary evaporation. After the complete removal of DCM from the RB-based GUMBOS, the sample was placed in a freezer for 24 h and then freeze-dried to eliminate any remaining water. The yields of the counterion exchange reactions were as follows: [TBP]_2_[RB]: 92.4 ± 0.8%, [TPP]_2_[RB]: 97.1 ± 3.1%, and [C_12_MIm]_2_[RB]: 87.2 ± 1.7%, indicating efficient synthesis with high reproducibility (Table S1).

### 2.4 Synthesis and Characterization of nanoGUMBOS

The series of RB-based nanoGUMBOS was synthesized by a simple reprecipitation method to form nanoparticles from a 10 mM RB-based GUMBOS stock solution in MeOH or DMSO. Briefly, a small aliquot of a MeOH/DMSO solution of each RB-based GUMBOS was rapidly introduced into a large volume of an aqueous cell medium (Dulbecco’s modified Eagle’s medium (DMEM), containing 10% fetal bovine serum) or water under ultrasonication. In this condition, a supersaturation of the hydrophobic RB-based GUMBOS is achieved in the aqueous system, leading to the precipitation of the RB-based GUMBOS into small particles. After that, the solution was placed on the bench and allowed to rest for a period of 15 min to allow the nucleation of the nanoparticles.

Once the incubation period concluded, DLS and S-TEM instruments were used to characterize the nanoGUMBOS. Dynamic light scattering was performed using a Malvern Zetasizer.^45^ The instrument was set to an automatic attenuator and had an associated standard operating procedure per GUMBOS absorbance value and refractive index. Each compound was scanned at 25 °C for 10 s each scan, and the system automatically adjusted the scans needed per run. S-TEM was employed via the use of a JEOL-SEM 7600F instrument, with a vacuum pressure of 1.9E^–4^ Pa, electron beam at 30,000 V, and amplified current of 9 A, with each micrograph content being produced at 8000× magnification. ImageJ software was used for the measurements of electron micrograph images. The polydispersity index from the TEM micrograph was calculated using eq 1.

1 $$\mathrm{PDI}=\frac{\mathrm{size}\;\mathrm{deviation}\left(\sigma \right)}{\mathrm{size}\;\mathrm{mean}\left(\mu \right)}$$

### 2.5 Water–Octanol Partition Experiments

The effective hydrophobicity of RB-based GUMBOS was determined through octanol/water partition experiments.^31,35,46–50^ Briefly, a standard calibration curve was constructed by using water-saturated octanol, and the absorbance of RB-based GUMBOS was measured at 562 nm (Figure S1). Equilibrium samples were prepared in triplicate by mixing an RB-based GUMBOS octanol solution at a specific concentration with an equal volume of water. These equilibrium solutions were stirred for 24 h, after which the absorbance of the octanol layer was measured, and the concentration of RB-based GUMBOS was determined using the calibration curve. The concentration of RB-based GUMBOS in the water layer was calculated by difference, and the octanol–water partition coefficients were calculated using eq 2.

2 $$K_{\mathrm{ow}}=\frac{{\left[\mathrm{GUMBOS}\right]}_{\mathrm{octanol}}}{{\left[\mathrm{GUMBOS}\right]}_{\mathrm{water}}}$$

### 2.6 *In Vitro* Parallel Artificial Membrane Permeability Assay (PAMPA)

The blood–brain barrier (BBB) permeability of RB-based GUMBOS was assessed using the PAMPA plate system assay (BD Biosciences, Bedford, MA).^51,52^ Briefly, 100 μL of specified concentration RB-based GUMBOS solution in DMSO was added into the donor plate, and 150 μL of phosphate saline buffer 1× (PBS 1×) solution was added into the receiving plate. The plate was incubated for 24 h at 25 °C, 1 atm, and in a dark environment. The absorbance spectra of the donor and receiver solutions are taken using a UV–vis instrument at a dilution of 8000 times the original state. The permeability of the GUMBOS was calculated using eqs 3 and 4.

3 $$C_{x}=\frac{V_{A}\times V_{D}}{\left(V_{A}+V_{D}\right)\mathrm{AREA}\times \mathrm{TIME}}$$

4 $$P=C_{x}\times \left(-\ln\frac{A_{\mathrm{acceptor}}}{A_{\mathrm{equilibrium}}}\right)$$

### 2.7 Confocal Microscopy

HEK293 or A549 Cells were collected from a T-25 culture flask following a standard cell culture harvesting procedure. A549 cells were diluted in cell culture media to a concentration of roughly 1,35,000 cells/mL. From this, the cells were loaded into individual chambers on an eight-chamber microscope slide, with a cell count per chamber of roughly 60,000 cells. This slide was then placed in an incubator (37 °C and 5.0% CO_2_ conditions) for 24 h before treating. When the treatment began, the original cell culture media was removed from the cells, and a compound/media mixture was added to each well. A control group of only cell culture media/DMSO was prepared and applied to one chamber in the slide, with the others consisting of treatment groups, following a serial dilution from 10 μM down to 0.15625 μM. After applying the treatment, the plate was returned to the incubator for 24 h. Following the incubation period, the cells were fixed using a 4% formaldehyde solution in the PBS buffer for 15 min at room temperature. After 15 min, the cells were washed three times with PBS. Then, to prepare the cells for staining, 0.5% Triton X-100 in PBS was applied to the cells for 15 min at room temperature. This was followed by three washes with PBS again. A 3% bovine serum albumin in PBS buffer was added to the cells for 60 min to block potential nonspecific binding sites for later 4′,6-diamidino-2-phenylindole (DAPI) staining. Another three washes with PBS were applied to the cells, and the cells were stained with a 1 μg/mL solution of DAPI and a mounting solution.

The slides were then mounted, and confocal imaging was observed in a blue channel with an emission maximum of 461 nm using a Zeiss confocal laser scanning microscope, LSM-710, and an inverted Axio Observer Z1. The imaging method employed fluorescence microscopy for nuclear staining visualization, allowing for an accurate quantification of viable cells based on intact nuclear morphology. ImageJ software was used to count the cell nuclei in each image. To avoid background signal, the software was set to only include nuclei of 20 μm^2^ or larger. Viability quantification was performed by counting intact, properly stained nuclei and expressing results as a percentage of control values, with error bars representing standard error of the mean (SEM) from triplicate experiments. Statistical analysis was performed using unpaired *t*-tests with significance set at *p* < 0.05. Each nanoGUMBOS treatment was compared independently to control within the same cell lines, representing planned pairwise comparisons rather than exploratory testing. Given this, each nanoGUMBOS formulation that reduces cell viability will be assessed for an untreated cell line. Comparisons are made within discrete experimental groups for each cell line; thus, the unpaired *t*-test is employed. Dose–response curves and IC_50_ values were generated using a four-parameter logistic regression model implemented in Python (v3.12) within Jupyter Notebook (Anaconda distribution). Curve fitting was performed with the SciPy package (version 1.13), and figures were prepared using Matplotlib (v3.9). The rationale for selecting A549 (lung carcinoma) and HEK293 (noncancerous embryonic kidney) cell lines was based on their well-characterized nature and widespread use in cancer research, representing a relevant cancer model and a noncancerous control, respectively. The use of embryonic kidney cells may not perfectly represent normal adult tissue responses, which is a limitation of this study, but will allow for the selectivity index to be produced for the most potent nanoGUMBOS synthesized. Selectivity will be calculated using eq 5.

5 $$\mathrm{SI}=\frac{\mathrm{cytotoxicity}\;\mathrm{of}\;A549\;\mathrm{cells}}{\mathrm{cytotoxicity}\;\mathrm{of}\;\mathrm{HEK}293\;\mathrm{cells}}$$

## 3 Results and Discussion

### 3.1 Synthesis and Characterization of GUMBOS

Three Rose Bengal-Based GUMBOS, [TBP]_2_[RB], [TPP]_2_[RB], and [C_12_MIm]_2_[RB], were synthesized through a simple metathesis reaction, and the sodium cation in the parent [Na]_2_[RB] salt was exchanged by an imidazolium and two phosphonium cations. Each organic salt complex synthesized showed high yields with low variance, demonstrating reproducible synthesis conditions. These syntheses did not require further synthesis steps or purification, as the removal of inorganic salt by-product was completed by multiple washes using deionized water. Scheme 1 shows each product’s theoretical structure, which was successfully isolated and characterized via the use of FT-IR, ESI-MS, UV–vis spectroscopy, and NMR.

**Scheme 1:**
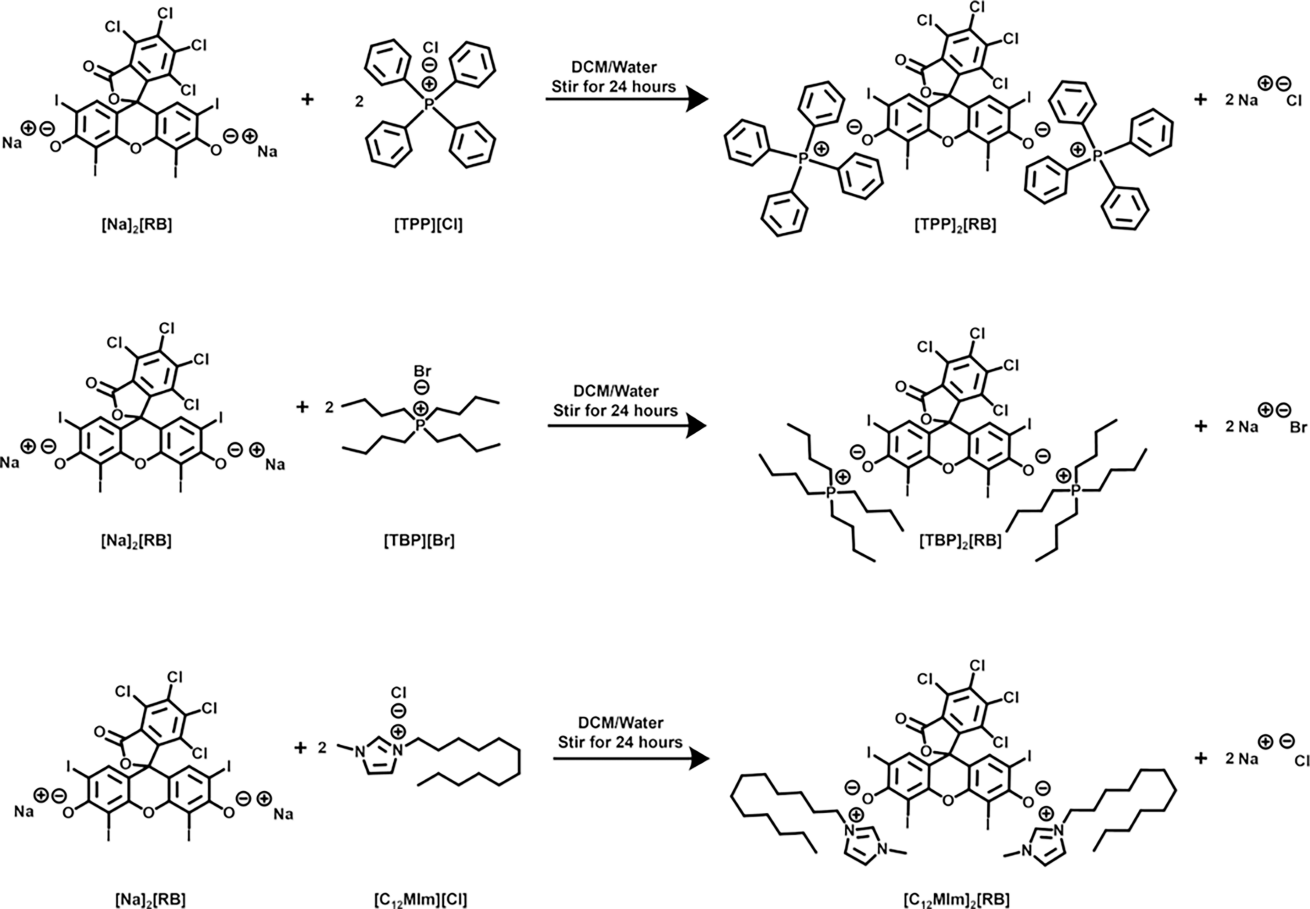
Synthesis Tetraphenylphosnium Rose Bengal [TPP]_2_[RB], Tetrabutylphosphonium Rose Bengal [TBP]_2_[RB], and 1-Methyl-3-dodecylimidalozium Rose Bengal [C_12_MIm]_2_[RB]

FT-IR analysis confirmed the retention of RB dye functional groups along with the incorporation of distinct organic cation structures (Figure S2). Each GUMBOS spectra maintain an ester line stretching visible in peak (C). For [TBP]_2_[RB], a sharp C–H peak (A) can be found at 3028 cm^–1^, indicating the addition of the organic cation containing butyl groups, in addition to the maintenance of C=C vibrations at peaks of 1552, 1437, and 1366 cm^–1^ from Rose Bengal’s aromatic rings. Further, [TPP]_2_[RB] contains a distinct aromatic C–H vibration at 744 cm^–1^ along with a distinct C=O stretch of Rose Bengal (B), indicating successful ionization of the phenyl-containing phosphonium. Finally, [C_12_MIm]_2_[RB] spectra exhibited a sharp and strong peak at 2980 cm^–1^ from C–H bending from the long carbon chain of the imidazolium ion alongside the consistent aromatic C=C stretches at 1563 cm^–1^.

ESI-MS analysis confirmed the effective ionization of the organic salts using a dual-pole analysis method. The experimental mass-to-charge ratios for each GUMBOS cation and [RB]^2–^ dianion, alongside low error between duplicate trials, indicate low degradation during synthesis (Table S2). In addition, the [C_12_MIm]^+^, [TPP]^+^, and [TBP]^+^ poles had very low deviance experimentally from the theoretically predicted mass-to-charge ratios. The mass spectrometry of each positive pole is provided in the Supporting Information (Figures S3–S5). NMR further confirmed the hydrogen and carbon structures for each parent structure and GUMBOS (Figures S6–S12). For [C_12_MIm]_2_[RB]: 1H NMR showed characteristic imidazolium protons at δ 8.7 (s, 1H, NCHN), alkyl chain protons at δ 4.1 (t, *J* = 7.2 Hz, 2H, NCH_2_), and the terminal methyl group at δ 0.9 (t, *J* = 6.8 Hz, 3H, CH_3_). For [TPP]_2_[RB]: aromatic phosphonium protons appeared at δ 7.6–7.8 (m, 20H, ArH). For [TBP]_2_[RB]: aliphatic protons were observed at δ 2.4 (m, 8H, PCH_2_) and δ 1.0 (t, *J* = 7.1 Hz, 12H, CH3). All spectra confirmed successful cation exchange while maintaining the RB structural integrity. Notably, distinct cationic peaks for aliphatic hydrogens and aromatic hydrogens were visible and accurately predicted by NMR and associated the effective ionization with the RB anion.

The spectral profile of Rose Bengal dye was found to consistently peak at 561.5 nm in MeOH. To ensure that spectroscopic properties are maintained, UV–vis spectroscopy was used to confirm chromophore excitation maxima at 561 nm, and subsequently fluorescence spectra of the excitation at 561 nm and emission maxima at 583 nm were recorded for the parent dye [Na]_2_[RB], and the GUMBOS [TBP]_2_[RB], [TPP]_2_[RB], and [C_12_MIm]_2_[RB] (Figure 1). All RB-based GUMBOS exhibited congruent spectra excitation and emissions, some with lower intensities, like [TBP]_2_[RB], compared with the parent dye spectrum. This indicates that the RB motif is the predominant chromophore species responsible for the spectral properties. The functionality of the optical characters for each GUMBOS confirms the potential anticancer effects for photodynamic reactivity within cancer cells.

**Figure 1:**
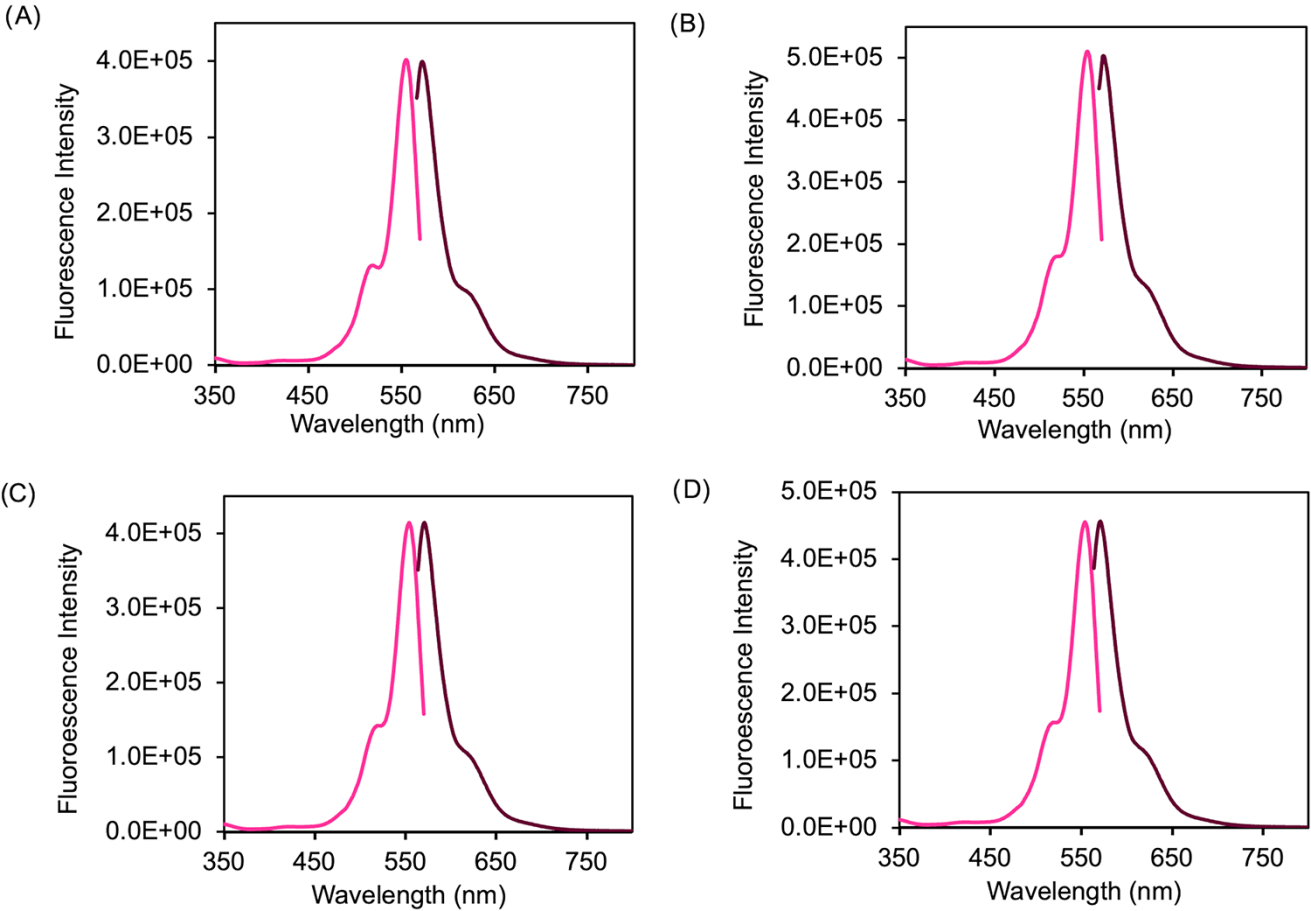
Excitation (pink) and emission (dark pink) spectra for (A) [Na]_2_[RB], (B) [TBP]_2_[RB], (C) [TPP]_2_[RB], and (D) [C_12_MIm]_2_[RB] GUMBOS.

### 3.2 Hydrophobicity Assessment

The influence of the cation identity on the GUMBOS hydrophobicity was assessed by a series of octanol–water partitioning experiments by UV–vis absorbance at a wavelength of 562 nm. The need for tunable hydrophobicity is to ensure that nanoparticle formation is effective through the described reprecipitation technique.^15,53,54^ The hydrophobic character of each compound assessed was interpreted and statistically analyzed (Table 1). Each compound exhibited hydrophobic character. [Na]_2_[RB] exhibited the highest hydrophobic nature with a partition coefficient of 4.5, indicating a strong affinity for the organic phase, with low variance indicating a reproducible and sensitive baseline for comparison. [TPP]_2_[RB] had the highest hydrophobic character among the synthesized GUMBOS, with a partition coefficient of 2.8, followed by [C_12_MIm]_2_[RB] with 2.5, and finally [TBP]_2_[RB] with 2.2. The structural correlation between log*P* values reveals that [TPP]_2_[RB] and [C_12_MIm]_2_[RB] show similar hydrophobic characteristics (log*P* values of 0.5 and 0.4, respectively), which can be attributed to the aromatic phenyl groups in [TPP] and the extended alkyl chain in [C_12_MIm], both contributing to lipophilic interactions. This tunability through counterion selection demonstrates the versatility of the GUMBOS approach for optimizing drug properties. The structures with more aromatic carbons had higher hydrophobicity in the set of synthesized GUMBOS, which aligns with previous literature for synthesizing hydrophobic GUMBOS and reprecipitation of nanoGUMBOS. Although the series of GUMBOS showed a decrease in hydrophobicity, the synthesis of nanoparticles is predicted to be more efficient due to the reducing planar structure of the RB salt. This is due to the predicted aggregation from stacking of the intercalating motif in RB, whereas the organic cations reduce this potential aggregation due to steric hindrance. These trials had slightly higher ranges of variance, but all confirmed the hydrophobic nature of the organic salt complexes. [Na]_2_[RB] had a higher hydrophobic character, consistent with the smaller sodium cations facilitating less aqueous dispersion than the organic cation counterparts. Overall, the results indicate that all RB-based GUMBOS have the capacity to form nanoparticles through a reprecipitation method, which is essential for effective membrane permeation and activity within cancer cells. The hydrophobicity trends observed align with nanomedicine benchmarks, specifically considering the synthesis of organic-based nanoparticles without the assistance of micelle formation.

**Table 1:** Results from Octanol–Water Partition Constants with the Average and Standard Deviation of the *K*_o/w_ ^a^

**GUMBOS**	***K***_**o/w**_	**Log(*P*)**	**SD**	**RSD**	**LOD**	**LOQ**
**[Na]**_**2**_ **[RB]**	4.5	0.7	0.02	0.4	0.3	1.1
**[TBP]**_**2**_ **[RB]**	2.2	0.3	0.04	1.8	0.7	2.4
**[TPP]**_**2**_ **[RB]**	2.8	0.5	0.05	1.7	0.6	1.9
**[C**_**12**_ **MIm]**_**2**_ **[RB]**	2.5	0.4	0.08	3.2	1.5	4.8

a SD indicates standard deviation; RSD is relative standard deviation; LOD is the limit of detection; and LOQ is the limit of quantification.

### 3.3 Synthesis and Characterization of nanoGUMBOS

Hydrophobic RB-based GUMBOS were fabricated into nanoGUMBOS using the direct reprecipitation technique. The spontaneous formation of nanoparticles by which they were characterized occurred in type III deionized water from solutions of GUMBOS in DMSO. The resulting solutions were characterized using UV–vis spectroscopy, DLS, and S-TEM.

The spectral profiles of the nanoparticles were found to consistently peak at 560 nm in type III deionized water. To confirm if the spectroscopic properties are maintained, UV–vis spectroscopy was used to confirm that the excitation and fluorescence spectra were recorded for the parent dye [Na]_2_[RB] and the nanoGUMBOS [TBP]_2_[RB], [TPP]_2_[RB], and [C_12_MIm]_2_[RB] at 10 μM (Figure 2). All RB-based nanoGUMBOS exhibited congruent spectra, excitation, and emissions. This indicates that the formation of nanoparticles under ultrasonication does not affect the chromophore motif of RB, ensuring that effective reactivity is maintained when cells uptake the nanoGUMBOS.

**Figure 2:**
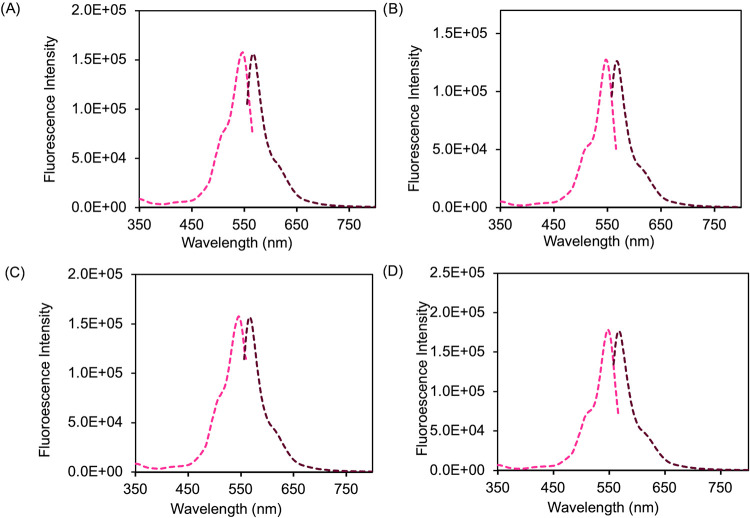
Excitation (pink) and emission (dark pink) spectra for (A) [Na]_2_[RB], (B) [TBP]_2_[RB], (C) [TPP]_2_[RB], and (D) [C_12_MIm]_2_[RB] nanoGUMBOS.

S-TEM revealed quasi-spherical morphologies for the newly synthesized nanoGUMBOS, with average diameters less than 100 nm. Dynamic light scattering measurements corroborated these findings, indicating monomodal intensity distributions. The nanoparticle size (<100 nm) is significant, as it falls within the optimal range for the enhanced permeation and retention (EPR) effect, facilitating passive tumor targeting through enhanced vascular permeability in tumor tissue. The dispersity of each nanoGUMBOS indicates a high level of uniformity within solutions (Table 2); however, TEM estimations indicate a higher level of disordered variance, which is attributed to a prolonged period of incubation during desiccation before period measurement.

**Table 2:** Nanoparticle Size Distribution from DLS and TEM Data from 10 μM Solutions of nanoGUMBOS

	**DLS**		**TEM**	
**GUMBOS**	**mean size** (nm)	**PDI**	**mean size** (nm)	**PDI**
**[Na]**_**2**_ **[RB]**	230.90 ± 17.89	0.58	268.62 ± 74.02	0.28
**[TBP]**_**2**_ **[RB]**	74.84 ± 4.01	0.45	81.94 ± 29.98	0.37
**[TPP]**_**2**_ **[RB]**	40.66 ± 3.33	0.22	45.33 ± 11.53	0.24
**[C**_**12**_ **MIm]**_**2**_ **[RB]**	59.87 ± 8.23	0.37	70.31 ± 16.43	0.23

The smallest nanoparticles synthesized were formed by [TPP]_2_[RB] at 40.66 nm by DLS, followed by [C_12_MIm]_2_[RB] and [TBP]_2_[RB], indicating that ion-pair hydrophobicity strongly influences the tendency of nucleation behavior toward the final particle dimensions (Figure 3). DLS shows that [Na]_2_[RB] nanoparticles are larger at 230.9 nm and exhibit a crystalline shared morphology from TEM, which can be explained by the higher aggregation tendency of the parent compound due to stronger intermolecular interactions between the planar RB anions and small sodium cations, resulting in less effective size control during reprecipitation (Figure 4).

**Figure 3:**
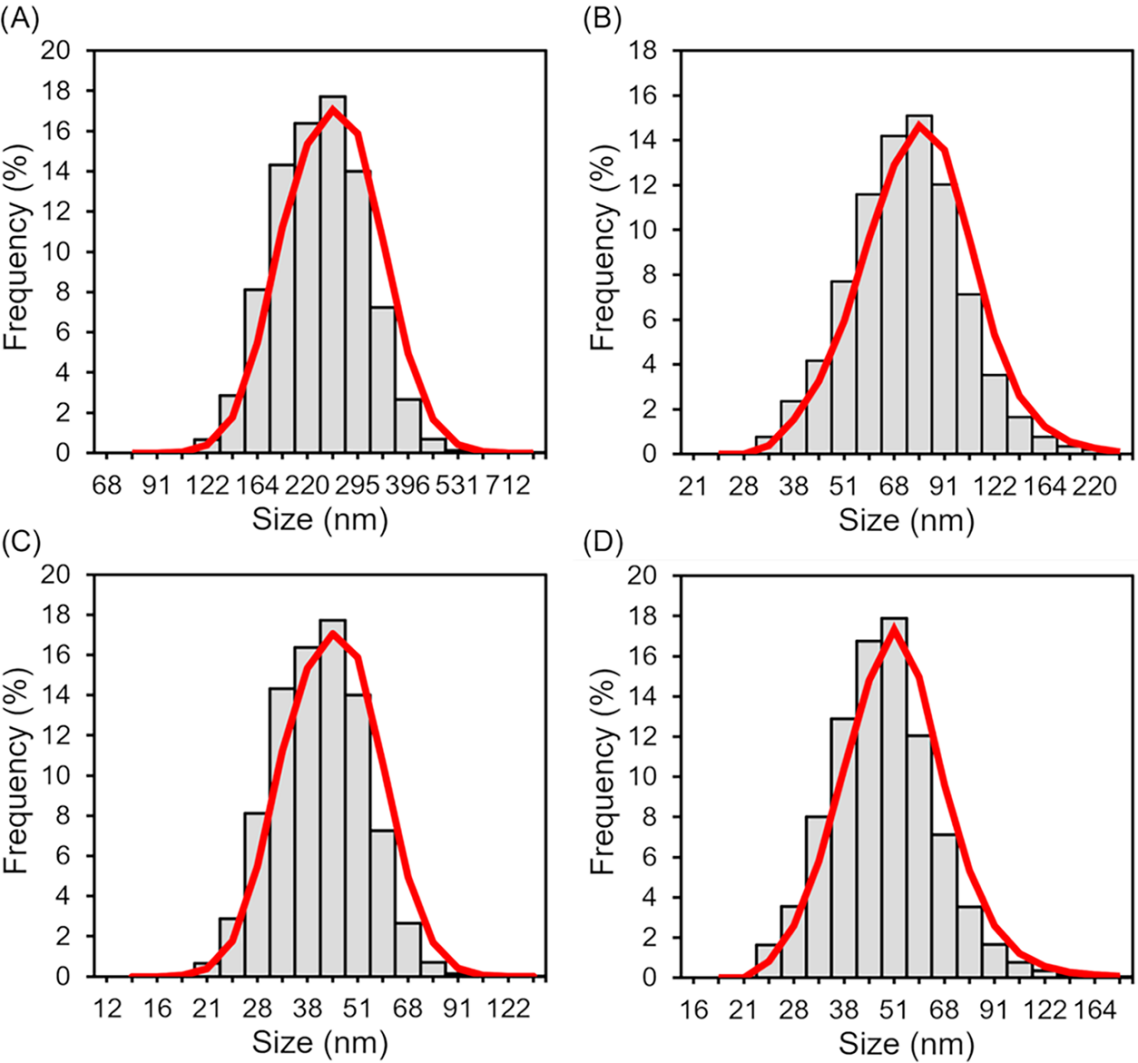
DLS size distribution for each nanoGUMBOS (A) [Na]_2_[RB], (B) [TBP]_2_[RB], (C) [TPP]_2_[RB], and (D) [C_12_MIm]_2_[RB].

**Figure 4:**
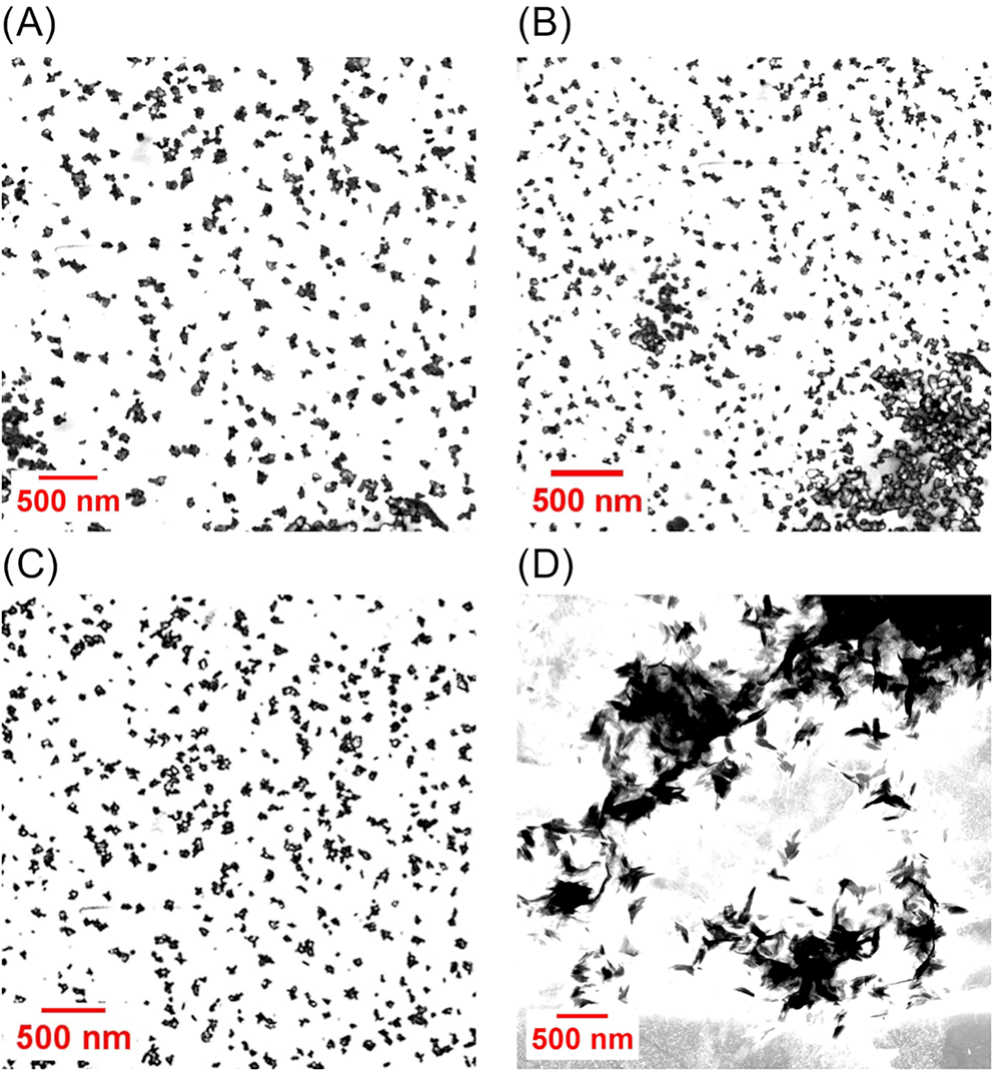
TEM micrographs for each RB-based nanoGUMBOS (A) [TBP]_2_[RB], (B) [TPP]_2_[RB], (C) [C_12_MIm]_2_[RB], and (D) [Na]_2_[RB].

ζ-Potential measures indicated moderately negative surface charges, which contributed to colloidal stability within aqueous and biological environments. This contrasts with the parent dye of RB in solution, with a negative ζ-potential of −18.4 mV, which is significantly different from the nanoGUMBOS synthesized. The negative ζ-potentials saw higher increases in negativity for [TPP]_2_[RB] and [C_12_MIm]_2_[RB] (Figure 5), indicating better potential within the cellular environment and an increased potential selectivity from homeostatic cell interactions. The enhanced negative ζ-potentials are attributed to the improved surface organization of RB anions in the presence of bulkier organic cations, leading to better charge distribution and colloidal stability. This is corroborated by the size distribution of the nanoparticles, with the parent dye solution exhibiting aggregation in solution.

**Figure 5:**
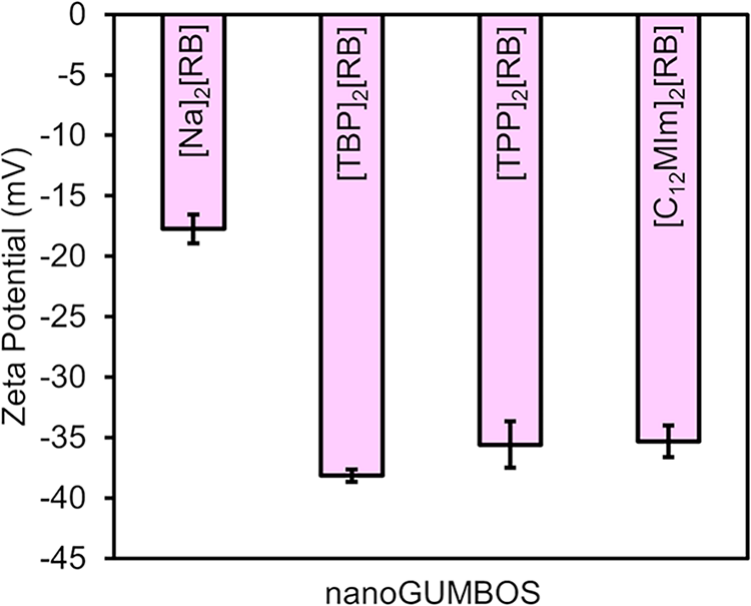
DLS ζ-potential for each Rose Bengal nanoGUMBOS by triplicate.

### 3.4 Cell Permeability Studies

To evaluate the potential for transcellular transport, a parallel artificial membrane permeability assay (PAMPA) was used to measure passive diffusion across a model blood–brain barrier (BBB) membrane. This model allows for the interpretation of the potential consideration for the anticancer effects of tumors formed in the central nervous system. As shown by Figure 6, all RB-based GUMBOS exhibited measurable permeability, with generally higher permeation velocities at lower concentrations. There was a statistically significant increase in the permeation velocity of three GUMBOS compared to the parent dye at both concentrations. Moreover, results indicated that dilution of each GUMBOS solution enhances the diffusion efficiency, likely due to reduced aggregation or micellar formation of the GUMBOS in solutions. Notably, this indicates a high potential for passive diffusion of the nanoGUMBOS into cells. An unpaired *t*-test was used for statistical analysis.

**Figure 6:**
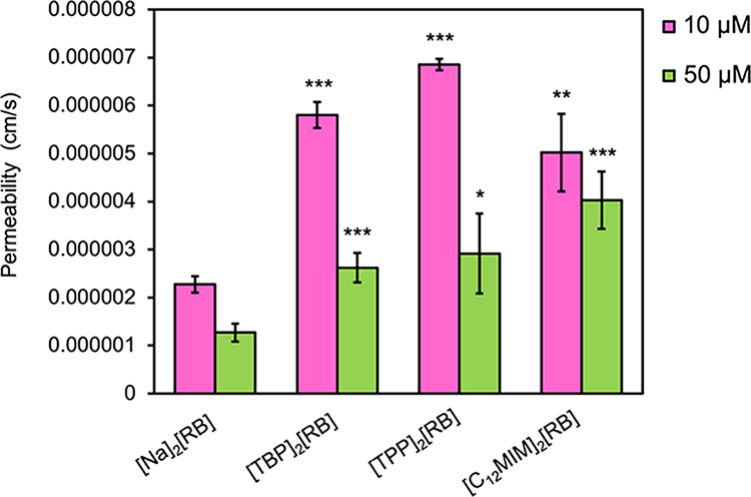
Permeability velocity of RB-based GUMBOS solutions at varying concentrations (*P < 0.05, **P < 0.001, ***P < 0.0001).

### 3.5 Confocal Microscopy Cell Viability Evaluation

To evaluate their therapeutic potential, the cytotoxic activity of RB-based nanoGUMBOS was assessed in A549 and HEK293 cells. The cells were treated for 24 h with 10 mM suspensions of each nanoGUMBOS formulation prior to the quantitation of intact DAPI-stained nuclei with confocal imaging in the dark (Figure 7). The fluorescence imaging suggests that cells treated with [TPP]_2_[RB] and [C_12_MIm]_2_[RB] showed altered nuclear morphology consistent with apoptotic processes, based on observable nuclear fragmentation and condensation, while [Na]_2_[RB] and [TBP]_2_[RB] exhibited minimal cellular changes. This indicates that the RB-based nanoGUMBOS induce cellular changes and reduce cellular viability through nonphotoactivated mechanisms. Future studies are proposed to investigate the mechanistic assays employed for apoptosis assessments for these nanoGUMBOS treatments and cell lines.

**Figure 7:**
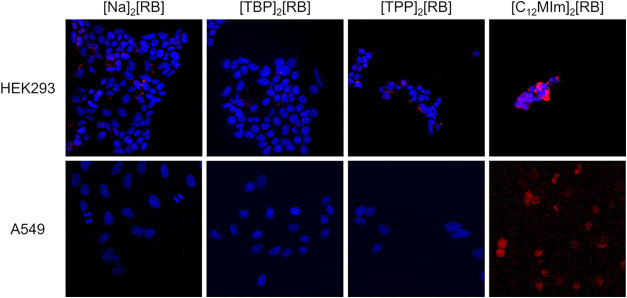
Confocal microscopy images of nuclei for A549 and HEK293 cell lines.

Statistical significance was assessed using unpaired *t*-tests comparing each treatment group to the respective control within the same cell line. There was no statistically significant difference between [Na]_2_[RB] and [TBP]_2_[RB] treatments (*p* > 0.05), whereas [TPP]_2_[RB] and [C_12_MIm]_2_[RB] treatments showed statistically significant reductions in cell viability compared to control (*p* < 0.05 for [TPP]_2_[RB]; *p* < 0.001 for [C_12_MIm]_2_[RB]). This indicates that treatment with [TPP]_2_[RB] and [C_12_MIm]_2_[RB] has anticancer potential, with reductions in cell viability below 50% (Figure 8). While both homeostatic and cancerous cell lines had reduced cell viability, A549 cancerous cells showed a significant difference in potency from the nanoGUMBOS solution, with an average viability decrease of 77.4% for the [C_12_MIm]_2_[RB] nanoGUMBOS treatment. [TPP]_2_[RB] had slightly weaker viability reductions at 57.2%. Both treatments for noncancerous HEK293 cells exhibited lower cell viability decreases at 63.1 and 42.7%, respectively. The selectivity indices (SI) were calculated as follows: [C_12_MIm]_2_[RB] SI = 2.1 and [TPP]_2_[RB] SI = 1.5. While these values indicate some degree of selectivity, we acknowledge that selectivity remains moderate, and further tuning of nanoGUMBOS is required to enhance the therapeutic window. The IC_50_ values for the most active compounds were determined: [C_12_MIm]_2_[RB] showed an IC_50_ of 2.2 μM against A549 cells (Figure S13). These substantial decreases in viability indicate that the primary treatment of [C_12_MIm]_2_[RB] nanoGUMBOS showed relative selectivity and potency toward cancerous cells. Following the successful identification of [C_12_MIm]_2_[RB] as the primary chemotherapeutic within this series of tunable hydrophobic GUMBOS.

**Figure 8:**
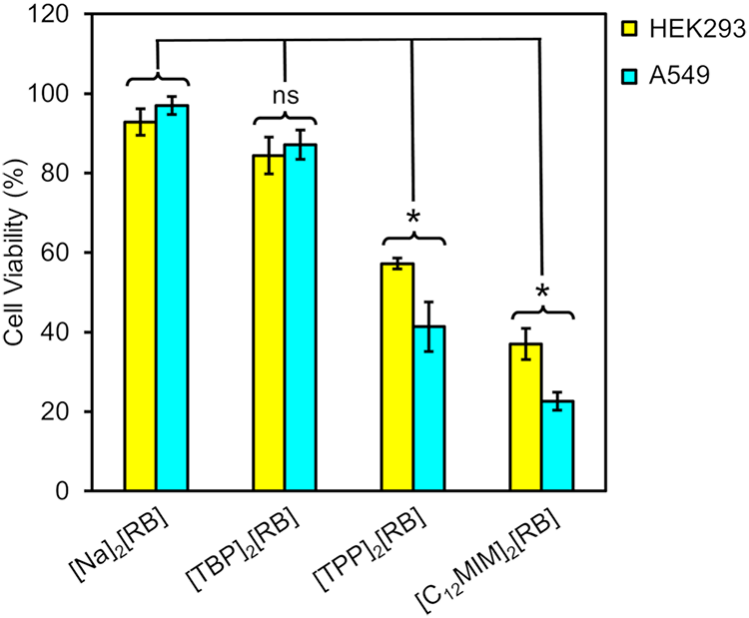
Cell viability for A549 lung cancer cells and HEK293 human embryonic kidney cells, each treated with 10 μM nanoGUMBOS solution (ns = not significant, *P < 0.05).

These results position [C_12_MIm]_2_[RB] nanoGUMBOS as a relevant therapeutic with a 50% inhibitory concentration, similar to those of other therapeutics. For comparison, one of the most widely investigated chemotherapeutic agents, cisplatin, has a varied IC50 between 2 and 15 μM and selectivity indices under 2 when tested on A549 cells.^55–57^ Doxorubicin, a type of anthracycline antibiotic, has an IC50 of 4.4 μM and a selectivity index of 1.5.^58^ It is also to highlight [C_12_MIm]_2_[RB] nanoGUMBOS improvement threshold when compared to [Na]_2_[RB] nanoGUMBOS. The parent dye exhibited limited cytotoxicity and poor selectivity (average viability of 86.7% for A549 cells at 10 μM). The nanoGUMBOS formulation represents a substantial improvement. This enhancement is derived from multiple factors: improved cellular uptake due to nanoparticle size (∼70 nm for [C_12_MIm]_2_[RB]), enhanced membrane permeability conferred by the lipophilic imidazolium cation (log*P* = 0.4), and favorable surface charge (ζ-potential ≈ −30 mV), which reduces aggregation and enhances colloidal stability in biological media. Thus, the similar therapeutic capabilities of nanoGUMBOS compared to those of traditional therapies set a precedent for further optimization for tuning of the salt complexes.

## 4 Conclusion

In this study, three hydrophobic Rose Bengal-based GUMBOS [TPP]_2_[RB], [TBP]_2_[RB], and [C_12_MIm]_2_[RB] were synthesized and characterized to explore their potential as tunable and selective chemotherapeutic agents. Spectroscopic analyses confirmed the successful ion exchange to form GUMBOS and the preservation of chromophore components. Spectrographic evidence, including representative ESI-MS, NMR, and chromatographic data, confirmed successful synthesis and structural integrity. Partition coefficient experiments demonstrated that the counterion identity strongly influenced the lipophilicity of each GUMBOS. From this, [TPP]_2_[RB], [TBP]_2_[RB], and [C_12_MIm]_2_[RB] nanoGUMBOS were successfully formed through the reprecipitation technique and exhibited a quasi-spherical morphology below 100 nm in diameter, with high uniformity and a negative ζ-potential of approximately −30 mV. PAMPA confirmed the ability of all three GUMBOS species to passively diffuse across lipid membranes, where low concentrations cause greater permeation velocities. *In vitro* cell viability was successfully assessed by confocal microscopy with HEK293 (noncancerous) and A549 (cancerous) cell lines. There was no statistically significant difference between [TBP]_2_[RB] and the parent dye [Na]_2_[RB]. There was a statistically significant decrease in cell viability for both cell lines with the treatments of [TPP]_2_[RB] and [C_12_MIm]_2_[RB], with the latter being extremely potent, with 22.6% cell viability for A549 cells. Further studies on this [C_12_MIm]_2_[RB] nanoGUMBOS elucidated a 50% inhibitory concentration (IC_50_) of 2.16 μM, and a selectivity index (SI) of 2.1. Each treatment had greater chemotherapeutic effects for the cancerous cell line compared to those of the homeostatic cell line, which had a higher cell viability. These findings suggest that Rose Bengal-based GUMBOS and nanoGUMBOS are tunable and slightly selective for cancerous cell lines. Future studies are planned to use Rose Bengal GUMBOS for protein binding assays. This would be done by optimizing the synthesis of long-chain cationic RB GUMBOS and nanoGUMBOS for binding to human and bovine albumin and further assessing hemolysis potential to see if increased bioavailability is achievable. Albumin-binding assays and subsequent half-life determination could be assessed by using fluorescence quenching or isothermal titration calorimetry. Additionally, mechanistic assays to interpret the pathways responsible for cell death are proposed to gauge the timeline of autophagy and apoptosis for cells treated with nanoGUMBOS. This could be done via flow cytometry-based Annexin V/PI double staining to distinguish early apoptosis (Annexin V^+^/PI^–^), late apoptosis (Annexin V^+^/PI^+^), and necrosis (Annexin V^–^/PI^+^).^59^ The generation of reactive oxygen species (ROS) could be quantified using fluorescent probes such as 2′,7′-dichlorofluorescein diacetate (DCFDA) or dihydroethidium (DHE) in tandem with further tuning of the nanoGUMBOS.

## Supplementary Material



## Notes

Percent yield for each GUMBOS synthesized in triplicate (Table S1). ESI-MS summary data for each GUMBOS, including theoretical and experimental mass-per-charge ratio (Table S2). Calibration curves (Figure S1). FT-IR spectra of the parent compounds and the corresponding RB-based GUMBOS. Positive pole and negative pole mass spectrum of [TBP]_2_[RB] (Figure S3). Positive pole and negative pole mass spectrum of [TPP]_2_[RB] (Figure S4). Positive pole and negative pole mass spectrum of [C_12_MIm]_2_[RB] (Figure S5). Proton and carbon NMR for [TPP]­[Cl] (Figure S6). Proton and carbon NMR for [TBP]­[Br] (Figure S7). Proton and carbon NMR for [C_12_MIm]­[Cl] (Figure S8). Proton and carbon NMR for [Na]_2_[RB] (Figure S9). Proton and carbon NMR for [TPP]_2_[RB] (Figure S10). Proton and carbon NMR for [TBP]_2_[RB] (Figure S11). Proton and carbon NMR for [C_12_MIm]_2_[RB] (Figure S12). IC_50_ curve for [C_12_MIm]_2_[RB] nanoGUMBOS treated on A549 lung carcinoma cells (Figure S13) (PDF)

## Abbreviations

ATP: adenosine triphosphate
BBB: blood–brain barrier
BSA: bovine serum albumin
[C_12_MIm]_2_[RB]: 1-dodecyl-3-methylimidazolium Rose Bengal
DAPI: 4′,6-diamidino-2-phenylindole
DCM: dichloromethane
DLS: dynamic light scattering
DMEM: Dulbecco’s modified Eagle’s medium
DMSO: dimethyl sulfoxide
ESI-MS: electrospray ionization-mass spectrometry
FT-IR: Fourier transform infrared spectroscopy
GUMBOS: group of uniform materials based on organic salts
MeOH: methanol
nanoGUMBOS: nanoparticles derived from GUMBOS
NMR: nuclear magnetic resonance
[Na]_2_[RB]: Rose Bengal disodium salt
PAMPA: parallel artificial membrane permeability assay
PBS: phosphate-buffered saline
RSD: relative standard deviation
LOD: limit of detection
LOQ: limit of quantification
[TBP]_2_[RB]: tetrabutylphosphonium Rose Bengal
TEM: transmission electron microscopy
[TPP]_2_[RB]: tetraphenylphosphonium Rose Bengal
UFLC: ultrafast liquid chromatography
UV–vis: ultraviolet–visible spectroscopy
