# Supplementary material for: Development of Tunable Rose Bengal-Based nanoGUMBOS as Potential Selective Chemotherapeutic Agents
Source: ACS Omega. 2025 Nov 13;10(46):55420–31. doi: 10.1021/acsomega.5c05765 (PMC12658820; doi:10.1021/acsomega.5c05765)
Supplement: Supplementary file 1 [file ao5c05765_si_001.pdf]

SUPPORTING INFORMATION

Development of Tunable Rose Bengal Based-nanoGUMBOS as Potential Selective Chemotherapeutic Agents

William J.A. Russell, Dylan J. Williams Daylon G. Douglas, Jannet Kocerha, Rocio L. Perez*

Center of Advance Materials Science (CAMS), Department of Biochemistry, Chemistry, and Physics, Georgia Southern University, Statesboro, GA, 30458

*Corresponding author: rperez@georgiasouthern.edu

Table S1. Percent Yield for each GUMBOS synthesized by triplicate.

	**Percent Yield (%)**
**$[TBP]_2[RB]$**	92.4 ± 0.8
**$[TPP]_2[RB]$**	97.1 ± 3.1
**$[C12MIm]_2[RB]$**	87.2 ± 1.7

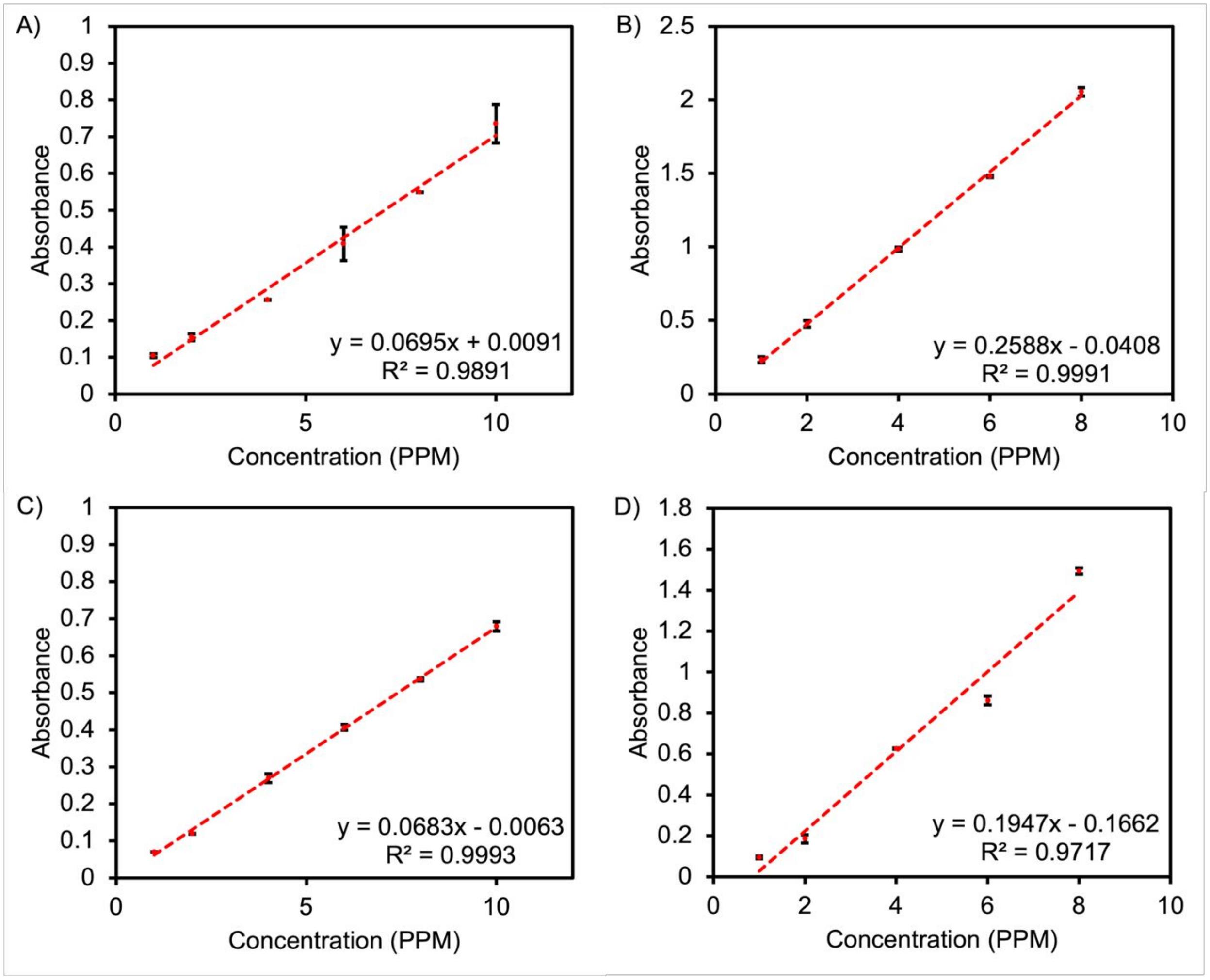

Figure S1. Calibration curves for A) $[TBP]_2[RB]$, B) $[TPP]_2[RB]$, C) $[C_{12}MIm]_2[RB]$, D) $[Na]_2[RB]$ nanoGUMBOS for octanol-water partition experiments.

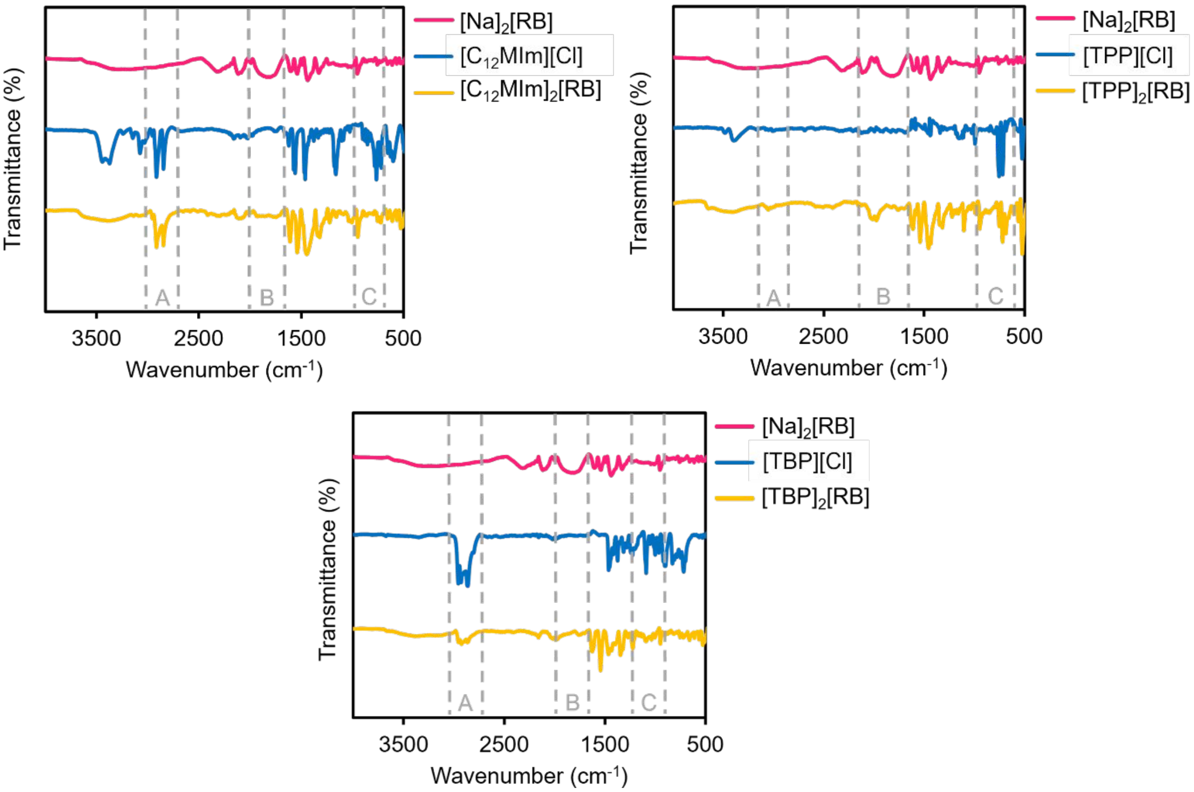

Figure S2: FT-IR spectra of the parent compounds and the corresponding RB based GUMBOS.

Table S2. ESI-MS summary data for each GUMBOS including theoretical and experimental mass-per-charge ration.

	**Cation (m/z)**		**Anion (m/z)**	
	**Theoretical**	**Experimental**	**Theoretical**	**Experimental**
**$[TBP]_2[RB]$**	259.26	259.11	971.48	971.12
**$[TPP]_2[RB]$**	339.13	339.34	971.48	971.76
**$[C_{12}MIm]_2[RB]$**	251.25	251.56	971.48	971.42

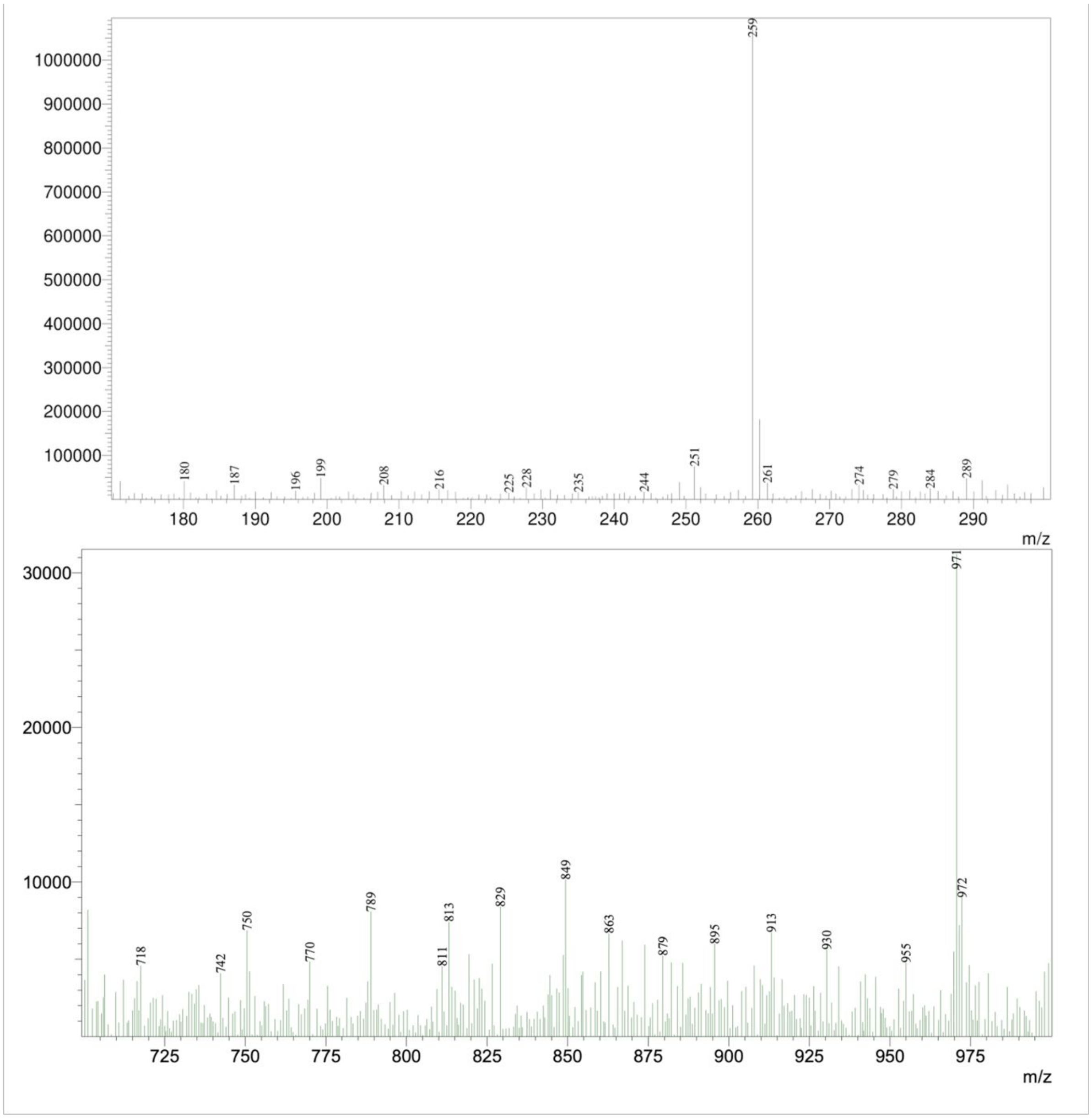

Figure S3. Positive pole and negative pole mass spectrum of [TBP]2[RB].

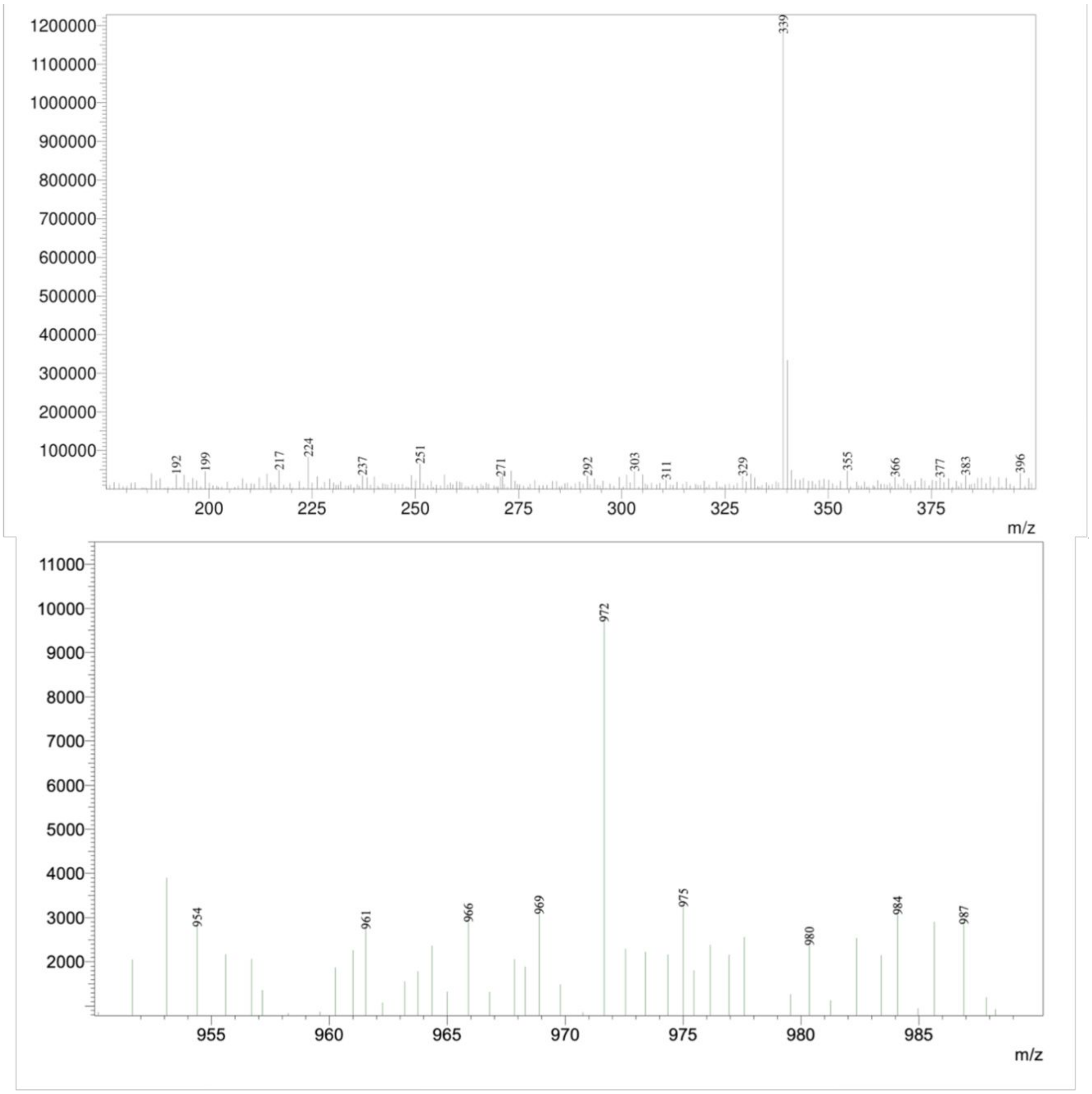

Figure S4. Positive pole and negative pole mass spectrum of $[TPP]_2[RB]$.

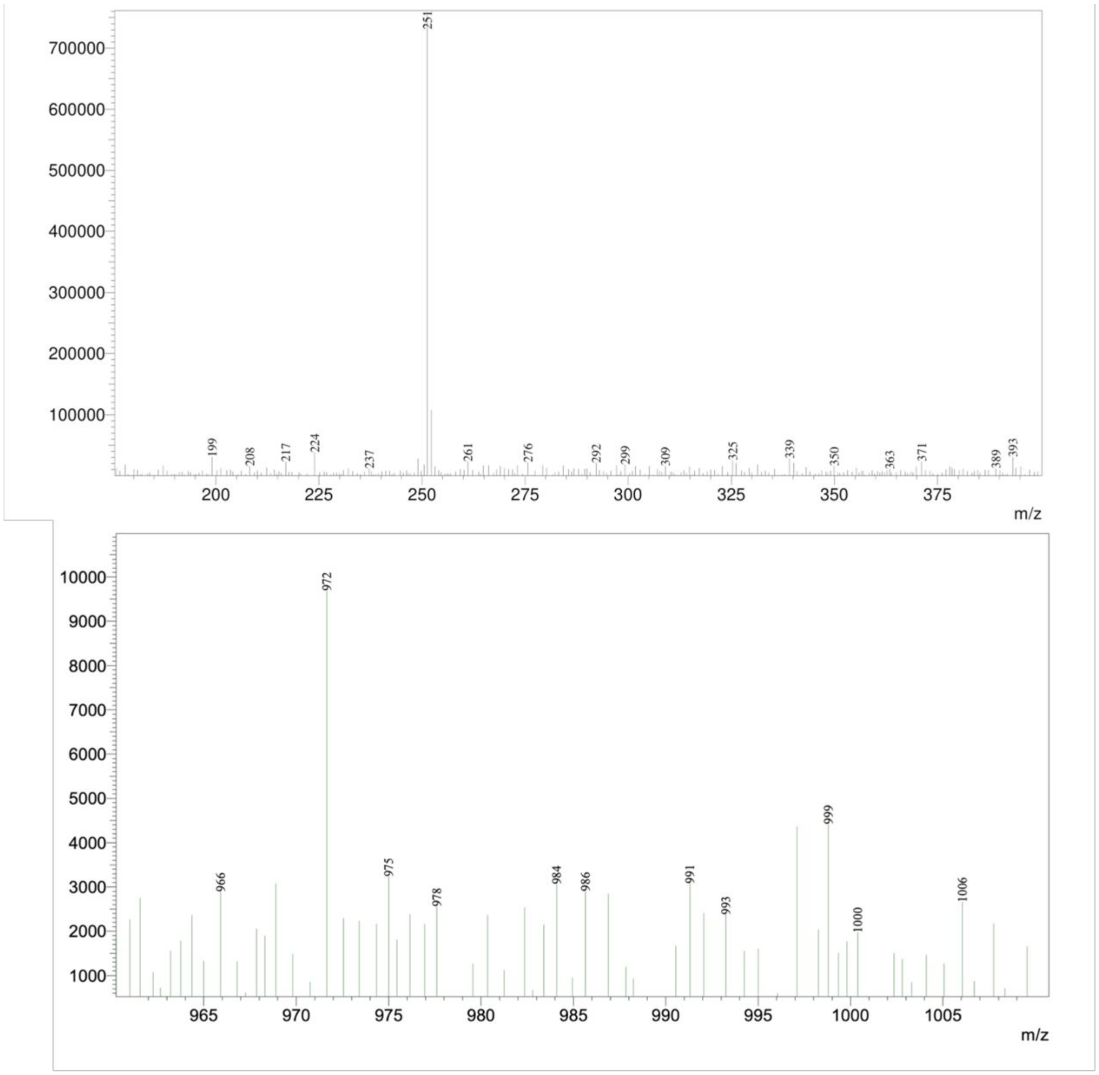

Figure S5. Positive pole and negative pole mass spectrum of $[C_{12}MIm]_2[RB]$.

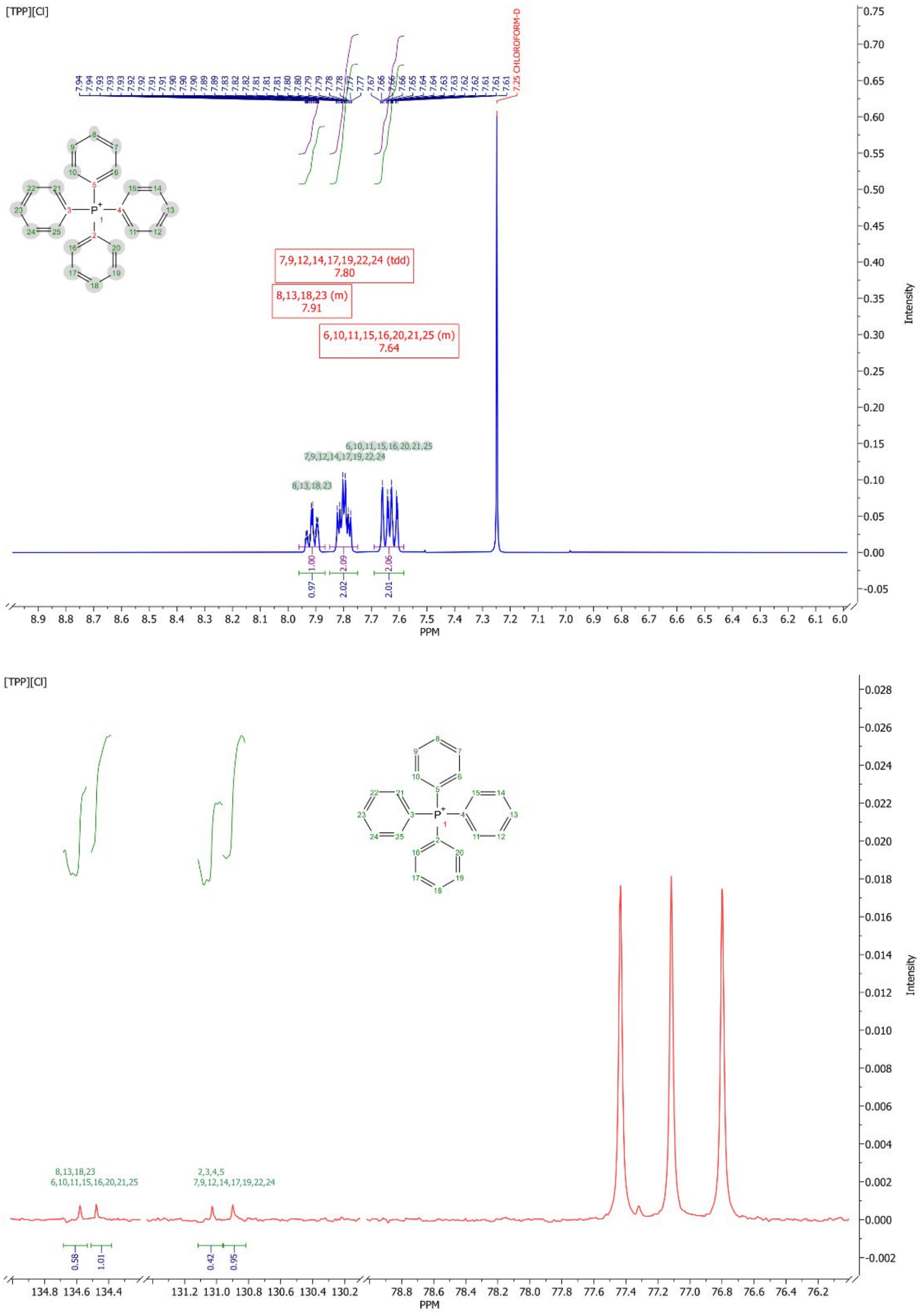

Figure S6: Proton and Carbon NMR for [TPP][Cl]. [TPP][Cl] was collected as a white powdery solid. ^{1}H NMR (400 MHz, Chloroform-D, ppm): δ = 7.93-7.89 (m, J = 7.91 Hz, 4H, Ph-H). 7.83-7.76 (tdd, J = 7.80 Hz, 8H, Ph-H). 7.67-7.59 (m, J = 7.64 Hz, 8H, Ph-H). ^{13}C NMR (100 MHz, Chloroform-D, ppm): δ = 134.6, 134.4, 131.0, 130.8.

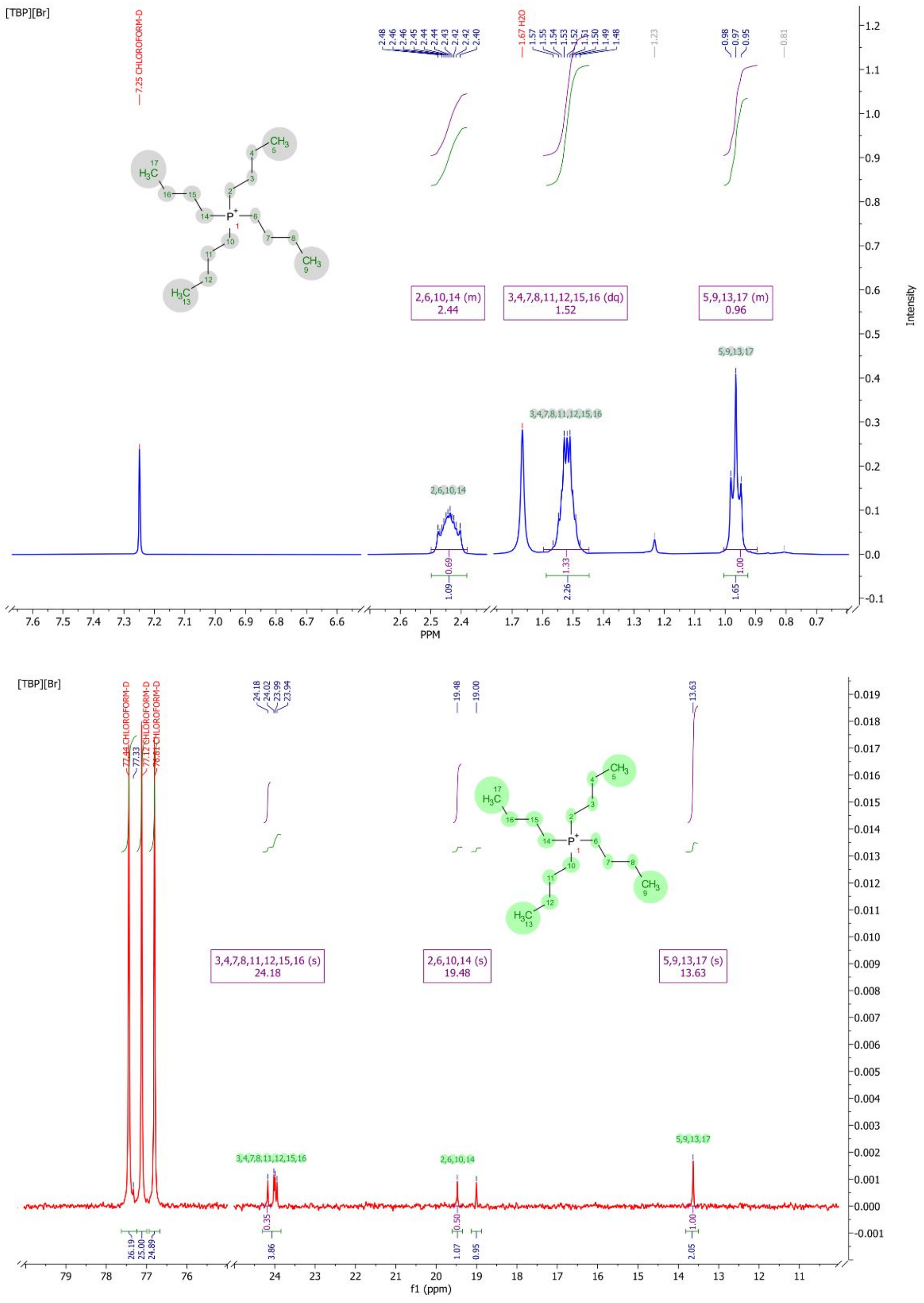

Figure S7: Proton and Carbon NMR for [TBP][Br]. [TBP][Br] was collected as a white crystalline solid. ^{1}H NMR (400 MHz, Chloroform-D, ppm): δ = 2.49-2.38 (m, J = 2.44 Hz, 8H, C-H). 1.59-1.44 (dq, J = 1.52 Hz, 16H, C-H). 1.03-0.94 (m, J = 0.80 Hz, 12H, C-H). ^{13}C NMR (100 MHz, Chloroform-D, ppm): δ = 24.18, 23.92 19.48, 13.63.

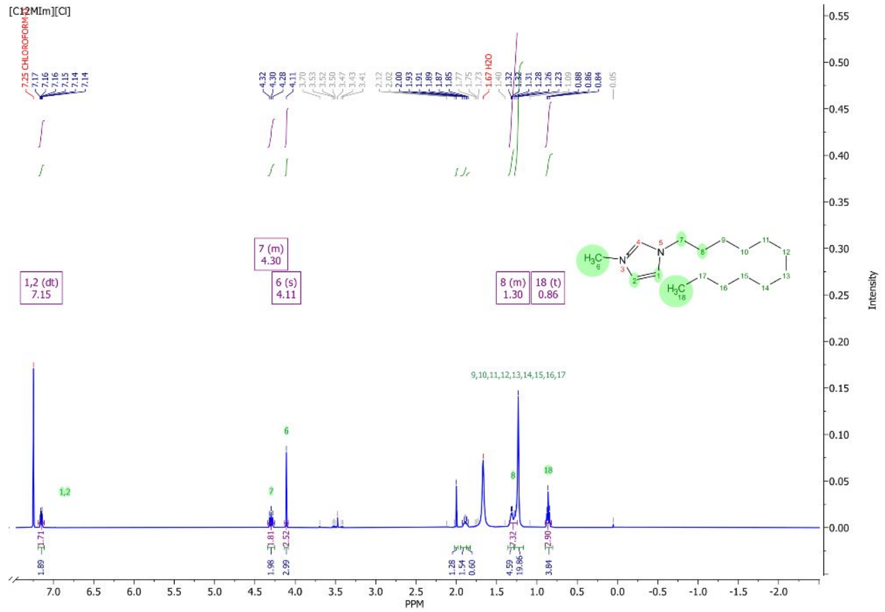

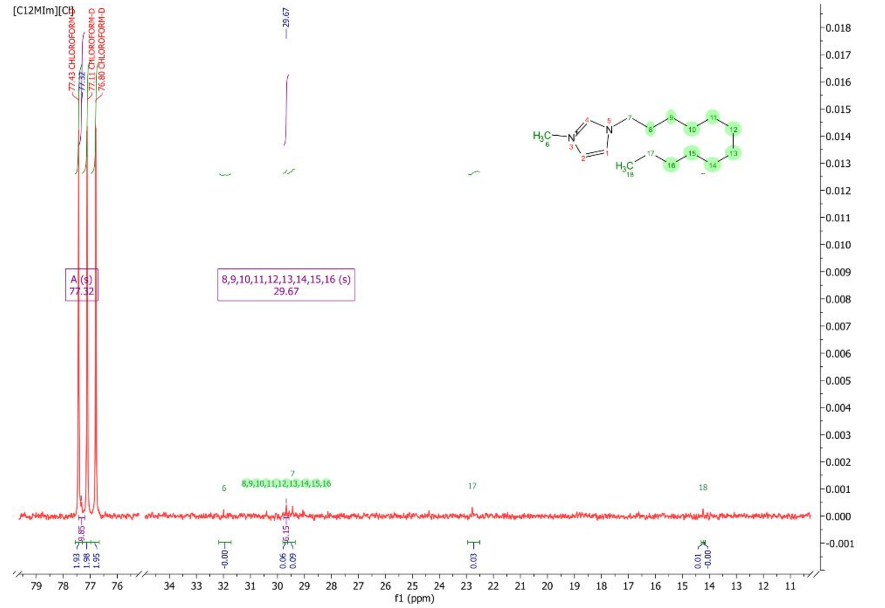

Figure S8: Proton and Carbon NMR for [C12MIm][Cl]. [C_{12}MIm][Cl] was collected as a yellow crystalline solid. ^{1}H NMR (400 MHz, Chloroform-D, ppm): δ = 7.18-7.11 (dt, J = 1.71 Hz, 2H, C=C-H). 4.33-4.27 (m, J = 1.81 Hz, 2H, C-H). 4.14-4.07 (s, J = 2.52 Hz, 3H, C-H). 1.37-1.29 (m, J = 7.32 Hz, 2H, C-H). 1.28-1.17 (s, J = 7.32 Hz, 18H, C-H). 0.90-0.80 (t, J = 2.90, 3H, C-H). ^{13}C NMR (100 MHz, Chloroform-D, ppm): δ = 31.99, 29.67, 29.43, 22.78, 14.26, peaks for carbon 1, 2, 4, and 7 not visible.

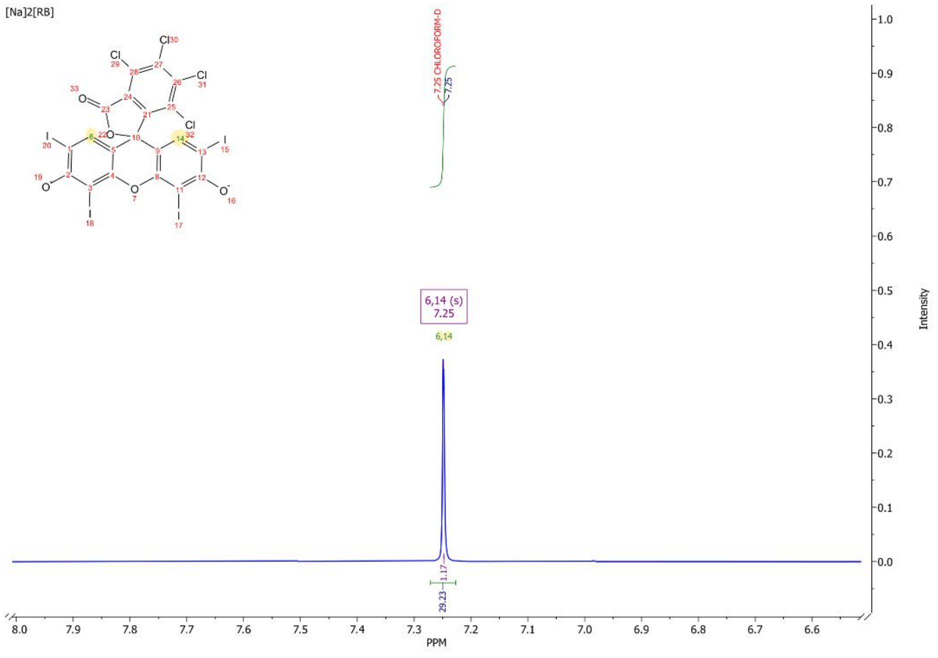

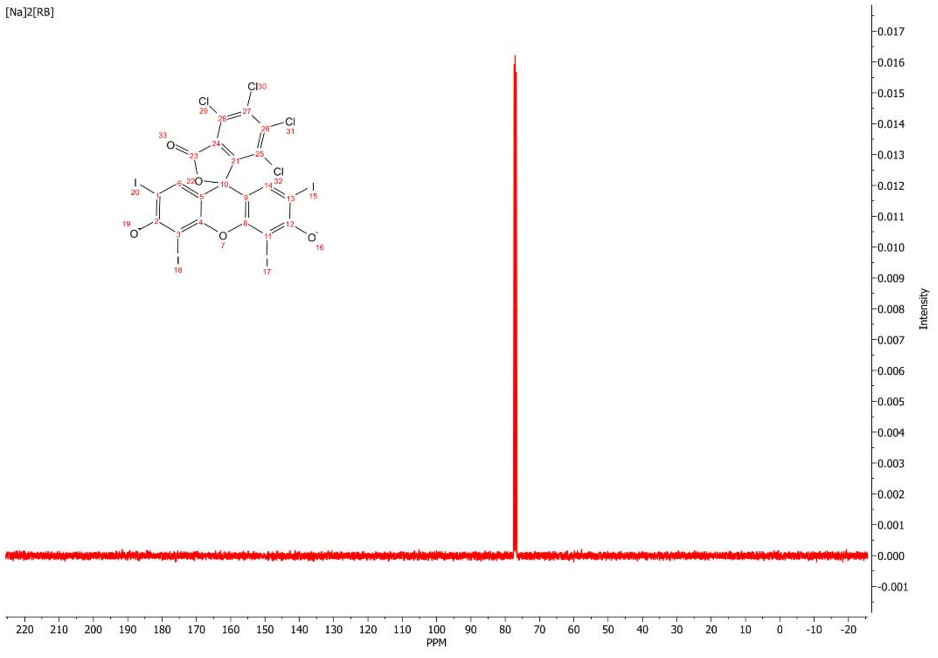

Figure S9: Proton and Carbon NMR for $[Na]_2[RB]$. $[Na]_2[RB]$ was collected as a dark pink powdery solid. ^1H NMR (400 MHz, Chloroform-D, ppm): δ = 7.26-7.23 (s, 2H, Ph-H) {overlapping with solvent peak}.^{13}C NMR (100 MHz, Chloroform-D, ppm): δ = no peaks were visible.

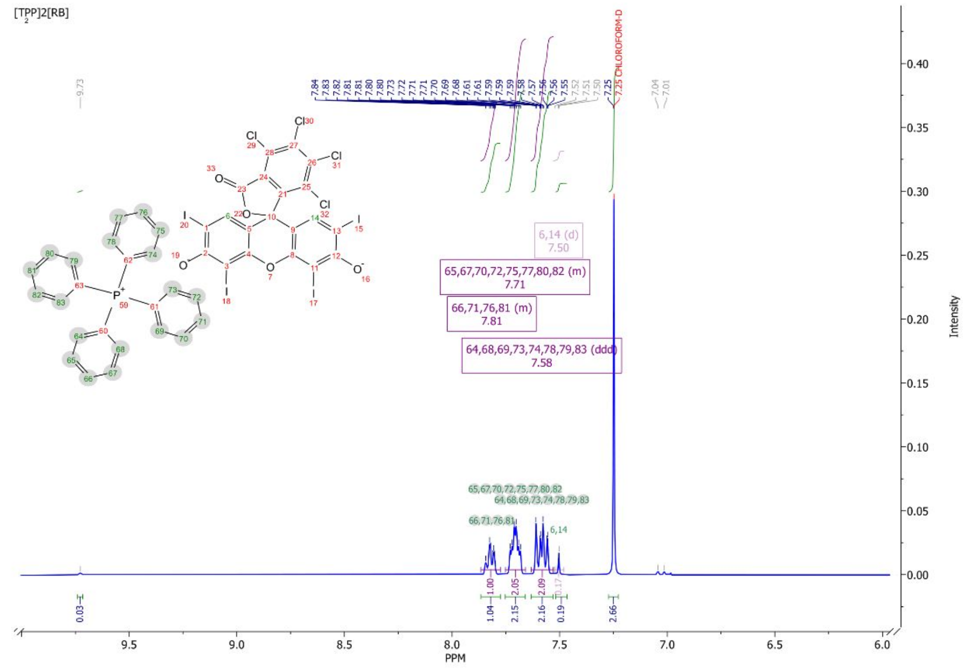

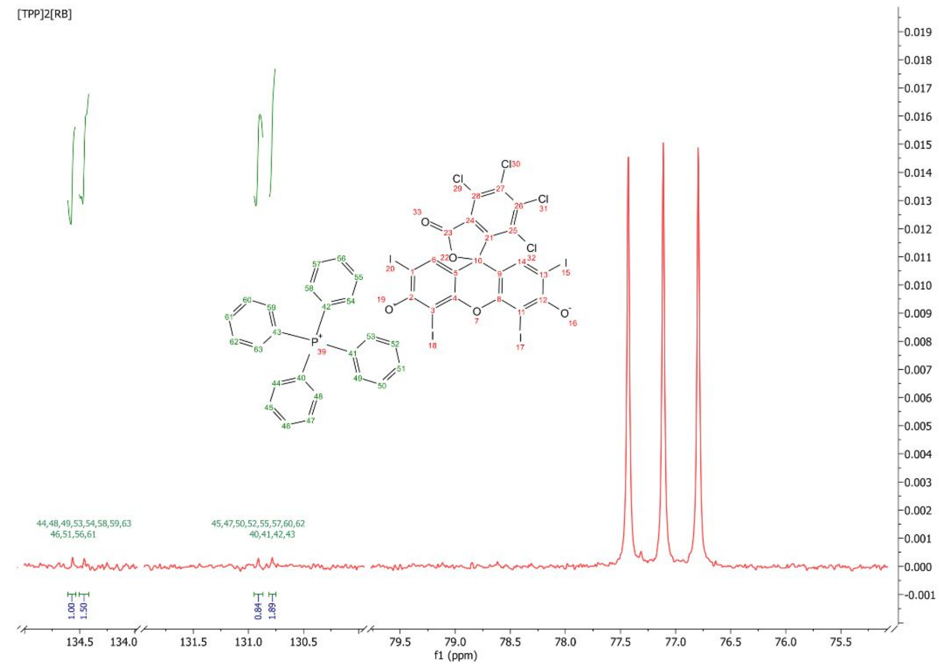

Figure S10: Proton and Carbon NMR for [TPP]$_2$[RB]. [TPP]$_2$[RB] was collected as a light pink powdery solid. δ = ^{1}H NMR (400 MHz, Chloroform-D, ppm): δ = 7.93-7.89 (m, J = 1.00 Hz, 4H, Ph-H). 7.83-7.76 (ddd, J = 2.05 Hz, 8H, Ph-H). 7.67-7.59 (m, J = 2.09 Hz, 8H, Ph-H). 7.51-7.48 (d, J = 0.17, 2H, Ph-H). ^{13}C NMR (100 MHz, Chloroform-D, ppm): δ = 134.55, 134.45, 130.92, 130.77; no rose bengal peaks were visible.

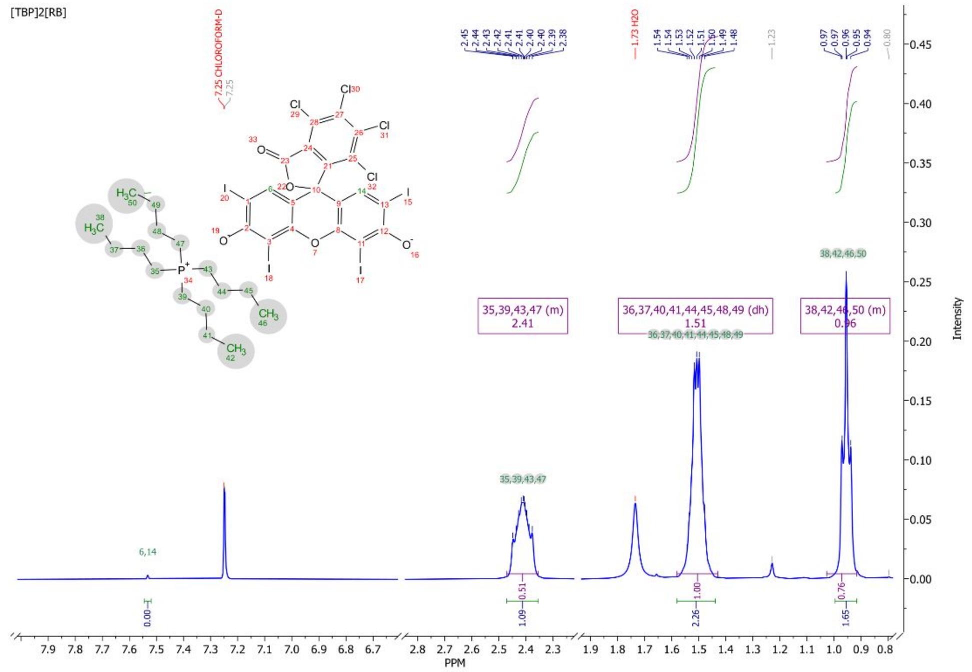

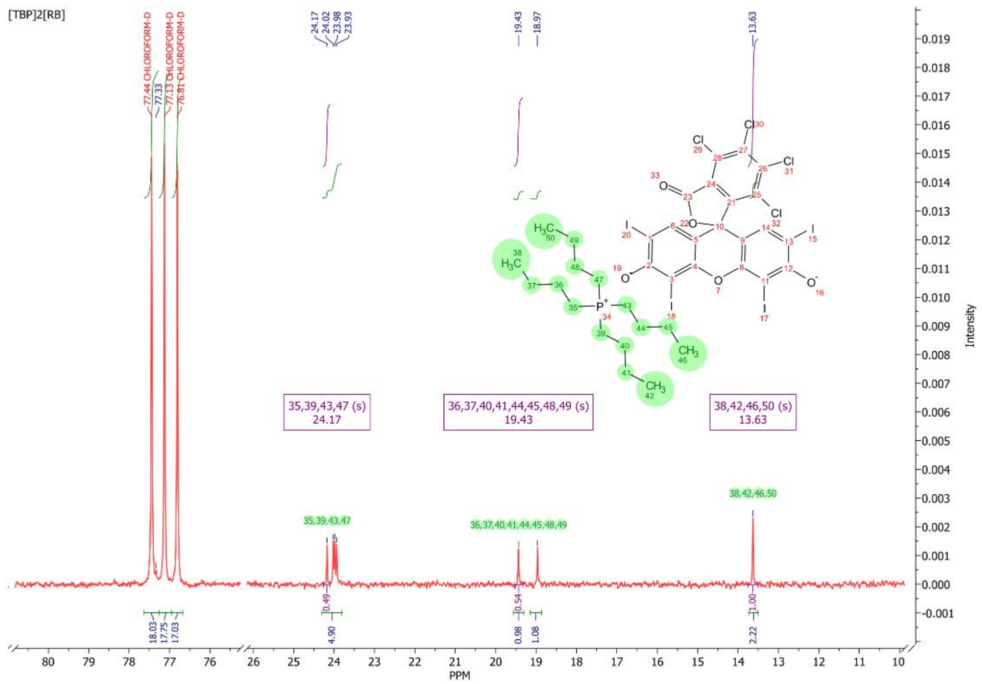

Figure S11: Proton and Carbon NMR for $[TBP]_2[RB]$. $[TBP]_2[RB]$ was collected as a dark pink crystalline solid. ^{1}H NMR (400 MHz, Chloroform-D, ppm): δ = 7.53-7.51 (s, 2H, Ph-H). 2.47-2.35 (m, J = 0.51 Hz, 8H, C-H). 1.58-1.42 (dh, J = 1.00 Hz, 16H, C-H). 1.02-0.96 (m, J = 0.76 Hz, 12H, C-H). ^{13}C NMR (100 MHz, Chloroform-D, ppm): δ = 24.17, 23.98 19.43, 13.62; no rose bengal peaks were visible.

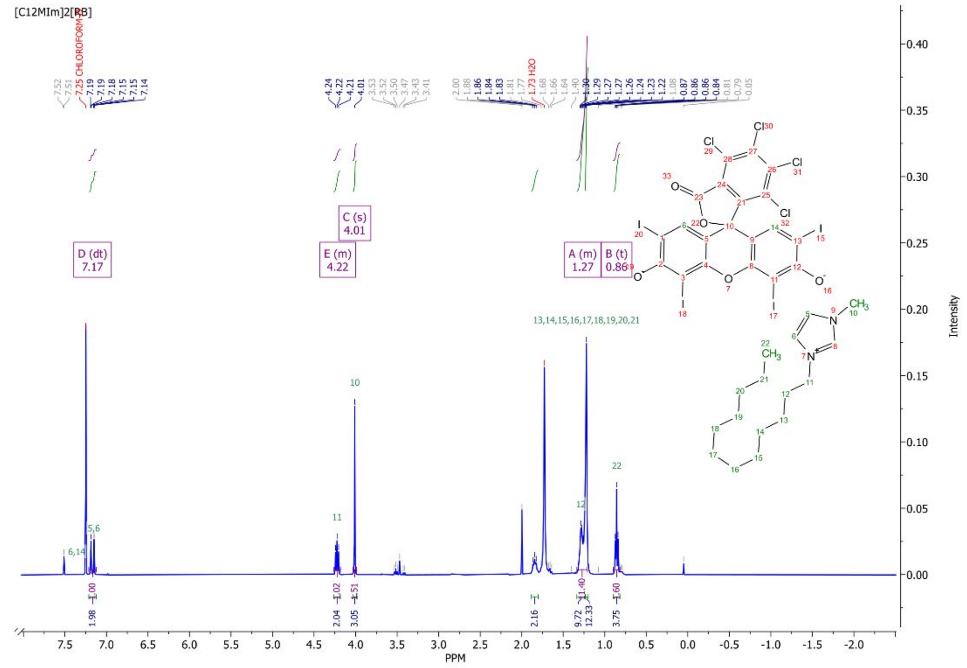

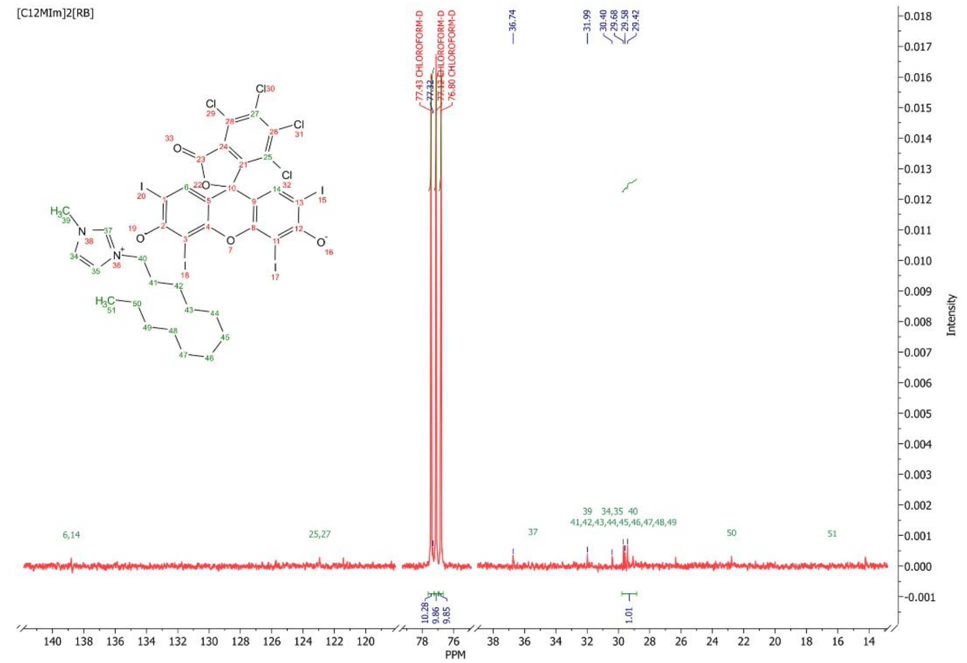

Figure S12: Proton and Carbon NMR for $[C_{12}MIm]_2[RB]$. $[C_{12}MIm]_2[RB]$ was collected as a light pink crystalline solid. ^1H NMR (400 MHz, Chloroform-D, ppm): δ = 7.52-7.49 (s, 2H, Ph-H). 7.19-7.15 (dt, J = 1.00 Hz, 2H, C=C-H). 4.24-4.20 (m, J = 1.02 Hz, 2H, C-H). 4.05-3.99 (s, J = 1.51 Hz, 3H, C-H). 1.31-1.25 (m, J = 1.4 Hz, 2H, C-H). 1.28-1.17 (s, J = 7.32 Hz, 18H, C-H). 0.85-0.83 (t, J = 1.60, 3H, C-H). ^{13}C NMR (100 MHz, Chloroform-D, ppm): δ = 138.81, 122.94, 36.72, 31.98, 30.40, 29.67, 29.42, 22.78, 14.23; some rose bengal peaks were not visible.

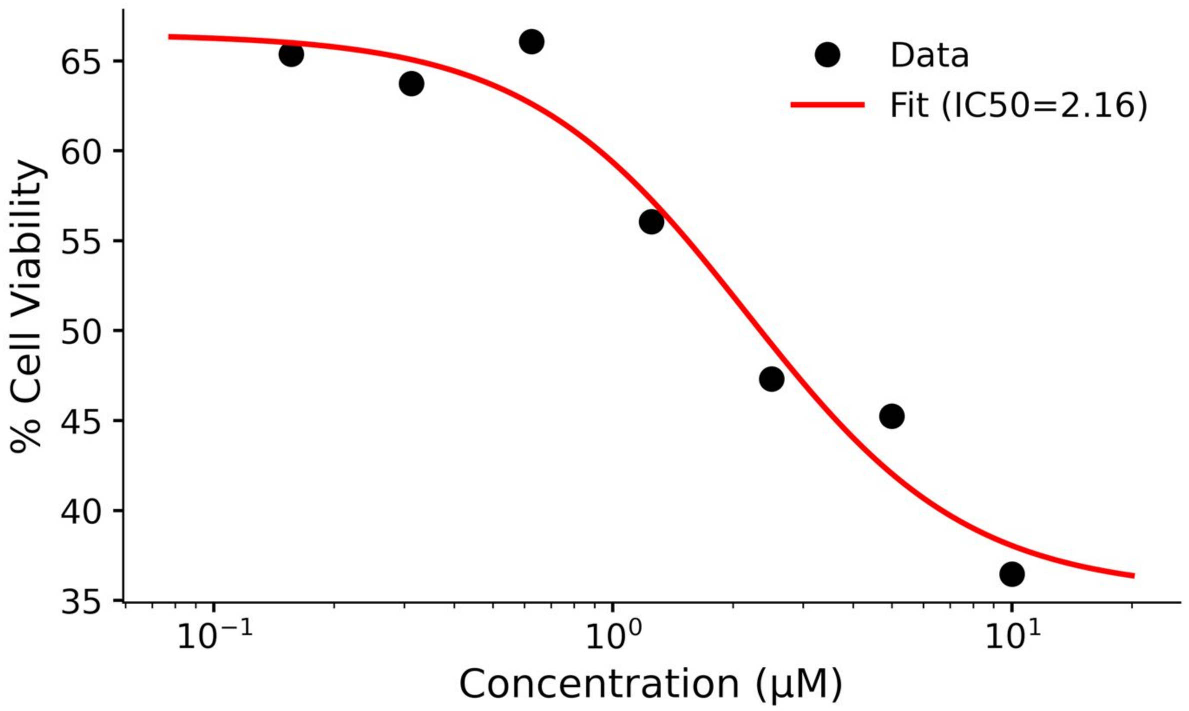

Figure S13: IC_{50} Curve for $[C_{12}MIm]_2[RB]$ nanoGUMBOS treated on A549 lung carcinoma cells.
